# Angiogenic factor AGGF1 blocks neointimal formation after vascular injury *via* interaction with integrin α7 on vascular smooth muscle cells

**DOI:** 10.1016/j.jbc.2022.101759

**Published:** 2022-02-22

**Authors:** Yubing Yu, Yong Li, Huixin Peng, Qixue Song, Xingwen Da, Hui Li, Zuhan He, Xiang Ren, Chengqi Xu, Yufeng Yao, Qing K. Wang

**Affiliations:** 1 Center for Human Genome Research, Key Laboratory of Molecular Biophysics of the Ministry of Education, and College of Life Science and Technology, Huazhong University of Science and Technology, Wuhan, Hubei, P. R. China; 2 College of Biotechnology, Guilin Medical University, Guilin, Guangxi, P. R. China

**Keywords:** angiogenic factor AGGF1, neointimal formation, restenosis after vascular injury, vascular smooth muscle cells, integrin α7, α5 and α8, AGGF1, angiogenic factor with G patch and FHA domains 1, BMS, bare metal stents, CAD, coronary artery disease, CVD, cardiovascular disease, DCB, drug-coated balloon, DES, drug-eluting stents, ERK1/2, extracellular signal-regulated kinases ½, MEK1/2, MAPK/ERK kinase ½, MI, myocardial infarction, PCI, percutaneous coronary interventions, VEGFA, vascular endothelial growth factor A, VSMCs, vascular smooth muscle cells

## Abstract

Angiogenic factor AGGF1 (AngioGenic factor with G-patch and FHA (Forkhead-Associated) domain 1) blocks neointimal formation (formation of a new or thickened layer of arterial intima) after vascular injury by regulating phenotypic switching of vascular smooth muscle cells (VSMCs). However, the AGGF1 receptor on VSMCs and the underlying molecular mechanisms of its action are unknown. In this study, we used functional analysis of serial AGGF1 deletions to reveal the critical AGGF1 domain involved in VSMC phenotypic switching. This domain was required for VSMC phenotypic switching, proliferation, cell cycle regulation, and migration, as well as the regulation of cell cycle inhibitors cyclin D, p27, and p21. This domain also contains an RDDAPAS motif via which AGGF1 interacts with integrin α7 (ITGA7), but not α8. In addition, we show that AGGF1 enhanced the expression of contractile markers MYH11, α-SMA, and SM22 and inhibited MEK1/2, ERK1/2, and ELK phosphorylation in VSMCs, and that these effects were inhibited by knockdown of ITGA7, but not by knockdown of ITGA8. *In vivo*, deletion of the VSMC phenotypic switching domain in mice with vascular injury inhibited the functions of AGGF1 in upregulating α-SMA and SM22, inhibiting MEK1/2, ERK1/2, and ELK phosphorylation, in VSMC proliferation, and in blocking neointimal formation. Finally, we show the inhibitory effect of AGGF1 on neointimal formation was blocked by lentivirus-delivered shRNA targeting ITGA7. Our data demonstrate that AGGF1 interacts with its receptor integrin α7 on VSMCs, and this interaction is required for AGGF1 signaling in VSMCs and for attenuation of neointimal formation after vascular injury.

Coronary artery disease (CAD) is the leading cause of death in the world, accounting for 15.9% of all types of death (1). Common treatment options for CAD include the minimally invasive percutaneous coronary interventions (PCI) and coronary artery bypass grafts (CABG). However, PCI is associated with vascular injury, which consequently results in re-occlusion or restenosis of the artery (2). Despite of advances of drug-eluting stents (DESs) that substantially reduced the rate of the restenosis, the rate remains about 10% (3). Therefore, much research effort is needed to further reduce the restenosis rate.

The restenosis is caused by neointimal formation after vascular injury, which is associated with the proliferation and migration of vascular smooth muscle cells (VSMCs) (4). We previously reported that AGGF1, an AnGiogenic Factor with G Patch and FHA Domains 1 (5), inhibited VSMC proliferation by promoting phenotypic switching of VSMCs to the contractile phenotype *in vitro* and *in vivo* in mice (6). Most interestingly, we showed that direct injection of purified AGGF1 blocked neointimal formation after vascular injury (6). The expression level of AGGF1 was dramatically reduced in carotid arteries at 14 and 28 days after vascular injury, and heterozygous *AGGF1*^*+/−*^ knockout (KO) mice showed increased VSMC proliferation and increased neointimal formation in carotid arteries after vascular injury (6). We showed that AGGF1 inhibited MEK1/2, ERK1/2, and ELK phosphorylation, promoted the formation of the myocardin/SRF/CArG-box complex involved in activation of VSMC contractile markers α-SMA and SM22, increased expression of cyclin D, and decreased expression of p21 and p27 (6). In a similar study, adenovirus-mediated *Aggf1* overexpression attenuated vascular injury by maintaining the contractile phenotype of VSMCs and stabilizing the SRF–myocardin complex (7). However, there are still many unanswered questions. For example, a receptor for AGGF1 on the surface of VSMCs is unknown. The molecular mechanism by which AGGF1 inhibits MEK1/2, ERK1/2, and ELK signaling needs to be identified.

AGGF1 is composed of 714 amino acids and contains a putative Forkhead-Associated (FHA) domain and a G-patch domain (5). FHA domains are known to be involved in phospho-dependent protein–protein interactions, and G-patch domains are known to be RNA-interacting modules (8). AGGF1 also contains an OCtamer REpeat (OCRE) domain, which was suggested to be involved in RNA metabolism and tumor necrosis factor (TNF)-activated signaling pathways (9). However, the functions of these domains in AGGF1 are unknown. More importantly, the domain responsible for VSMC functions and neointimal formation as well as MEK, ERK1/2, and ELK signaling remains to be identified.

Integrins constitute a large family of α/β-heterodimeric receptors that regulate multicellular organization and communication between the different cell types in the early metazoans (10). Integrins integrate the extracellular matrix with the intracellular cytoskeleton to mediate cell migration and adhesion (11). The RGD tripeptide (arginine–glycine–aspartic acid) is a structural recognition motif for cell surface integrins (12). In this study, we used deletion analysis to define an AGGF1 domain critical to VSMC phenotypic switching between amino acids 574 and 614, which contains an RDDAPAS motif with sequence homology to the RGD-binding motif for integrins. We then analyzed the three major integrins in VSMCs, integrin α5, α7, and α8. We showed that AGGF1 used integrin α7, but not α5 or α8, as a receptor on VSMCs, and suppressed neointimal formation after vascular injury *via* interacting with integrin α7 and regulating the phenotypic switching, proliferation and migration of VSMCs through the MEK-ERK1/2-ELK signaling pathway. The results provide fundamental understanding of a novel therapy based on AGGF1 to block neointimal formation and restenosis after vascular injury.

## Results

### Identification of an AGGF1 domain critical for phenotypic switching of VSMCs

We previously reported that AGGF1 inhibited neointimal formation and restenosis after vascular injury by promoting phenotypic switching of VSMCs from a synthetic state to the contractile state (6). In order to identify the critical AGGF1 domain involved in VSMC phenotypic switching, we created 12 deletion mutants by systematically truncating AGGF1 from the N-terminus and 13 deletion mutants from the C-terminus by every 50 amino acids (Fig. S1). All mutant AGGF1 proteins and WT AGGF1 were expressed in *E. coli*, and purified, and quality was ensured by analysis with SDS-PAGE. We then analyzed the effects of WT and mutant AGGF1 on phenotypic switching of MOVAS-1, a cell line for VSMCs, by examining the expression levels of contractile marker genes *MYH11*, *ACTA2* (encoding α-SMA), and *TAGLN* (encoding SM22). Quantitative RT-PCR analysis showed that the expression of *MYH11*, *ACTA2*, and *TAGLN* was significantly upregulated in MOVAS-1 by treatment with WT AGGF1 and deletion mutants AGGF1-C1 and AGGF1-C2 compared with control PBS; however, the effect got lost by deletion mutants AGGF1-C3 to AGGF1-C13 (Fig. S2, *A*–*C*). The data suggest that critical AGGF1 domain involved in VSMC phenotypic switching is located between AGGF1-C2 and AGGF1-C3, which corresponds to amino acids 564 and 614. Similar analysis with N-terminal deletion mutants indicates that the critical AGGF1 domain involved in VSMC phenotypic switching is located between AGGF1-N10 and AGGF1-N11, which corresponds to amino acids 574 and 624 (Fig. S2, *D*–*F*). Together, these data suggest that the critical AGGF1 domain involved in VSMC phenotypic switching is located between amino acids 574 and 614.

**Figure 1:**
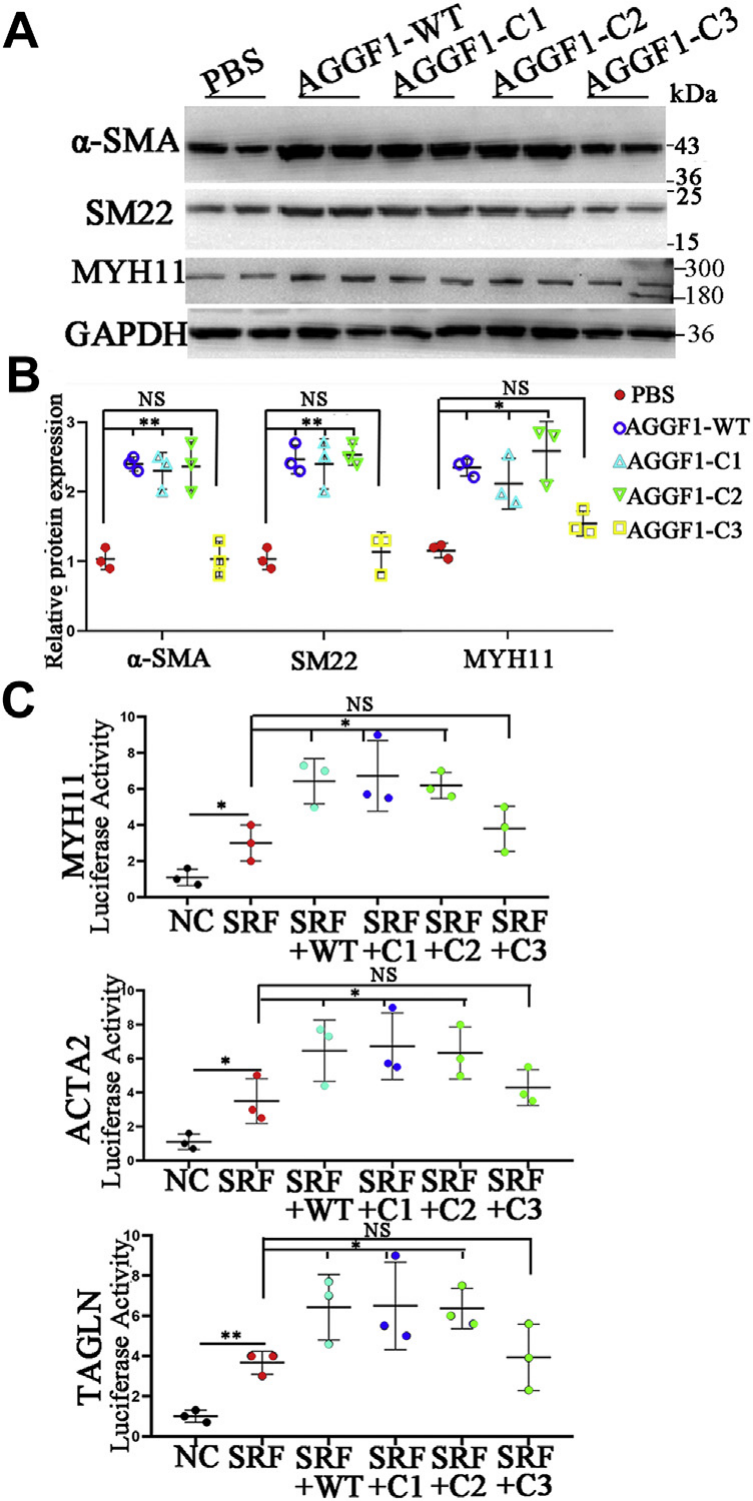
**Wild-type and mutant AGGF1-C1 and C2, but not AGGF1-C3, increase the expression of VSMC phenotypic switching markers α-SMA, SM22, and MYH11 in VSMCs.***A*, Western blot analysis for α-SMA, SM22, and MYH11. MOVAS cells were treated with 20 μl control PBS or 20 μl of 5 μg/ml wild-type AGGF1 (AGGF1-WT) or mutant AGGF1 (AGGF1-C1, AGGF1-C2 and AGGF1-C3) for 24 h, lysed, and used for Western blot analysis. *B*, quantification of Western blot images as in (*A*) (mean ± SD, one-way ANOVA with Dunnett test for multiple comparison; ∗*p* < 0.05, ∗∗*p* < 0.01, n = 3/group). *C*, luciferase assays showing that AGGF1-WT, AGGF1-C1 and AGGF1-C2 increased transcriptional activation of VSMC contractile marker genes encoding MYH11, a-SMA and SM22 in the presence of SRF (serum response factor), but the effects were not observed for AGGF1-C3. MOVAS cells were cotransfected with an expression plasmid for SRF together with a *MYH11*, *ACTA2*, or *TAGLN* promoter luciferase reporter gene with or without an expression plasmid for wild type or mutant *AGGF1*. Cells were lysed and used for luciferase assays 48 h after transfection. NC, empty vector. Data are shown as mean ± SD (one-way ANOVA with Dunnett test for multiple comparison; ∗*p* < 0.05, n = 3/group). NS, not significant. AGGF1, angiogenic factor with G patch and FHA domains 1; VSMCs, vascular smooth muscle cells.

We performed Western blot analysis for key AGGF1 deletion mutants to validate the findings from quantitative RT-PCR analysis. As shown in Figure 1, *A* and *B*, Western blot analysis showed that AGGF1-WT, AGGF1-C1, and AGGF1-C2 increased the protein levels of MYH11, α-SMA, and SM22 compared with PBS; however, the effect got lost by AGGF1-C3. The data confirm the findings from quantitative RT-PCR analysis in Fig. S2.

Luciferases assays with MYH11p-luc, ACTA2p-luc, TAGLNp-luc promoter-luciferase reporters containing the CArG box showed that overexpression of WT AGGF1, AGGF1-C1, and AGGF1-C2 stimulated SRF-mediated transcriptional activation of the *MYH11* promoter, *ACTA2* promoter, and *TAGLN* promoter; however, the effect got lost by AGGF1-C3 (Fig. 1*C*). The data further confirm the findings from quantitative RT-PCR and Western blot analyses.

**Figure 2:**
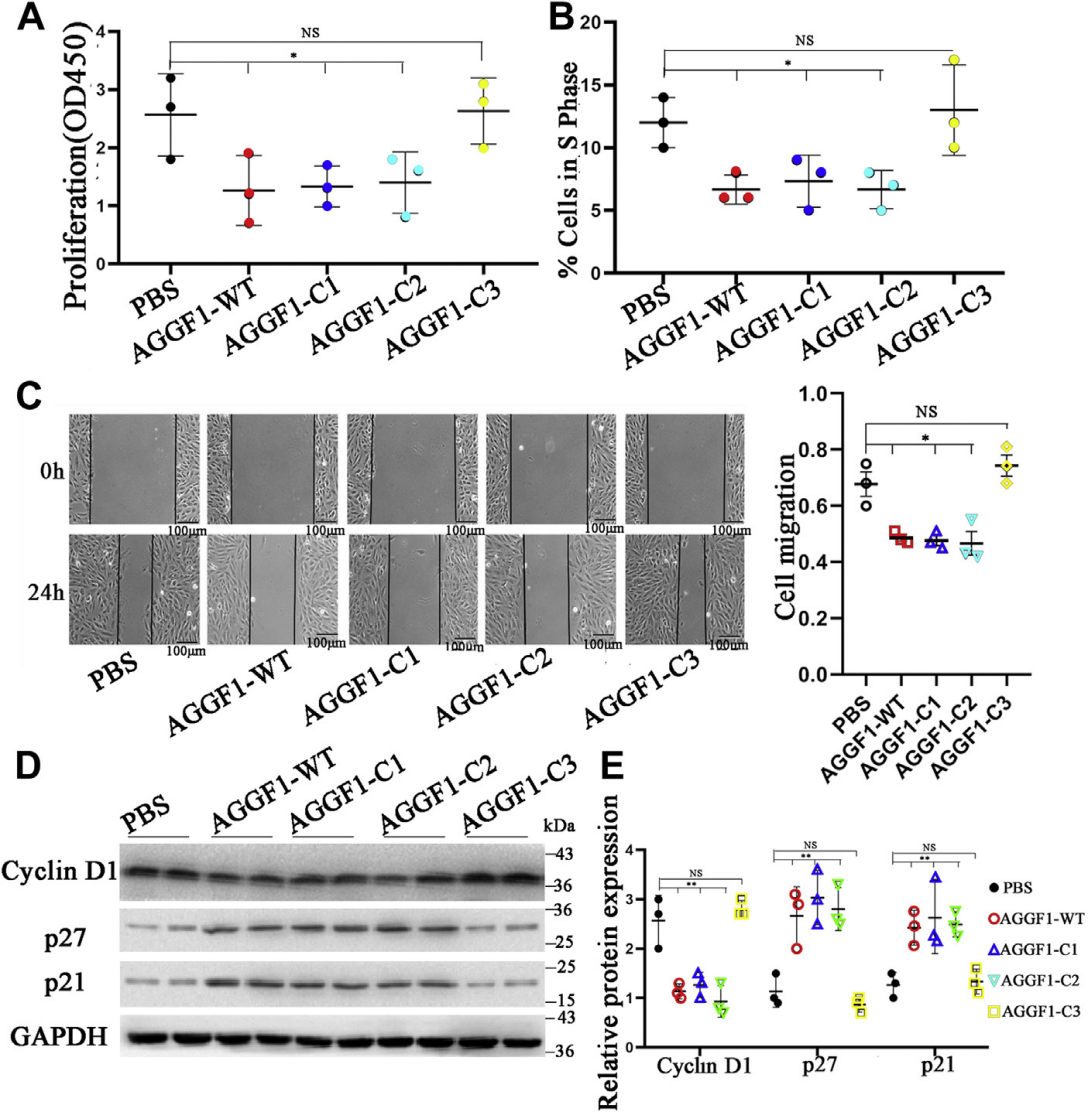
**Mutant AGGF1-C3 lost the effect of AGGF1 on cell proliferation, cell cycle regulation, and migration of vascular smooth muscle cells.***A* and *B*, MOVAS cells were incubated with 20 μl of wild-type AGGF1 (AGGF1-WT) (5 μg/ml) or mutant AGGF1 AGGF1-C1, AGGF1-C2, and AGGF1-C3 (5 μg/ml) *versus* 20 μl PBS control for 36 h (*A*) or 24 h (*B*–*D*), and used for cell proliferation assays (*A*) and cell cycle analysis (*B*). AGGF1-WT, AGGF1-C1, and AGGF1-C2 inhibited the proliferation of VSMCs, but this effect was not observed for AGGF1-C3. *A*, cell proliferation assays were performed with the CCK8 kit. Data are shown as mean ± SD (one-way ANOVA with Dunnett test for multiple comparison; ∗*p* < 0.05, n = 3/group). AGGF1-WT, AGGF1-C1 and AGGF1-C2, but not AGGF1-C3, significantly decreased the number of S-phase cells (*B*). Cell cycle analysis was performed and the number of S-phase cells was measured by flow cytometry. Data are shown as mean ± SD (one-way ANOVA with Dunnett test for multiple comparison; ∗*p* < 0.05, n = 3/group). *C*, AGGF1-WT, AGGF1-C1 and AGGF1-C2, but not AGGF1-C3, inhibited the VSMC migration in scratch-wound healing assays. MOVAS cells were cultured in a 6-well plate overnight, and a scratch was made on the bottom of wells. A total of 20 μl of wild type AGGF1 protein (AGGF1-WT) (5 μg/ml), mutant AGGF1 proteins AGGF1-C1, AGGF1-C2 and AGGF1-C3 (5 μg/ml) or PBS control was added. The cells were allowed to migrate for 24 h. The degree of cell migration was quantified and shown on the right. *D*, AGGF1-WT, AGGF1-C1, and AGGF1-C2, but not AGGF1-C3, inhibited expression of cyclin D and upregulated p27 and p21. MOVAS cells were treated as above, lysed, and used for Western blot analysis. *E*, quantification of Western blot images as in (*D*) (mean ± SD, one-way ANOVA with Dunnett test for multiple comparison; ∗∗*p* < 0.01, n = 3/group). NS, not significant. AGGF1, angiogenic factor with G patch and FHA domains 1; VSMCs, vascular smooth muscle cells.

The treatments with AGGF1-WT, AGGF1-C1, and AGGF1-C2 significantly inhibited the proliferation of MOVAS-1 cells (Fig. 2*A*) and decreased the cell numbers at the S phase during mitosis (Fig. 2*B*); however, the effect got lost by AGGF1-C3. As shown in Figure 2*C*, AGGF1-WT, AGGF1-C1, AGGF1-C2 treatments significantly inhibited VSMC migration; however, the effect got lost by AGGF1-C3. Western blot analysis was used to examine the expression levels of key cell cycle regulatory proteins cyclin D1, p27 and p21. AGGF1-WT, AGGF1-C1, and AGGF1-C2 decreased the expression level of cyclin D1 and increased the expression levels of p27 and p21; however, the effects got lost by AGGF1-C3 (Fig. 2*D*). Together, the data suggest that the AGGF1 domain involved in VSMC phenotypic switching (amin acids 564–614 between C2 and C3) is also involved in cell proliferation, migration, cell cycle regulation, and regulation of key cell cycle and proliferation genes in MOVAS-1.

### AGGF1 regulates phenotypic switching of MOVAS-1 *via* interaction with integrin α7

**Figure 3:**
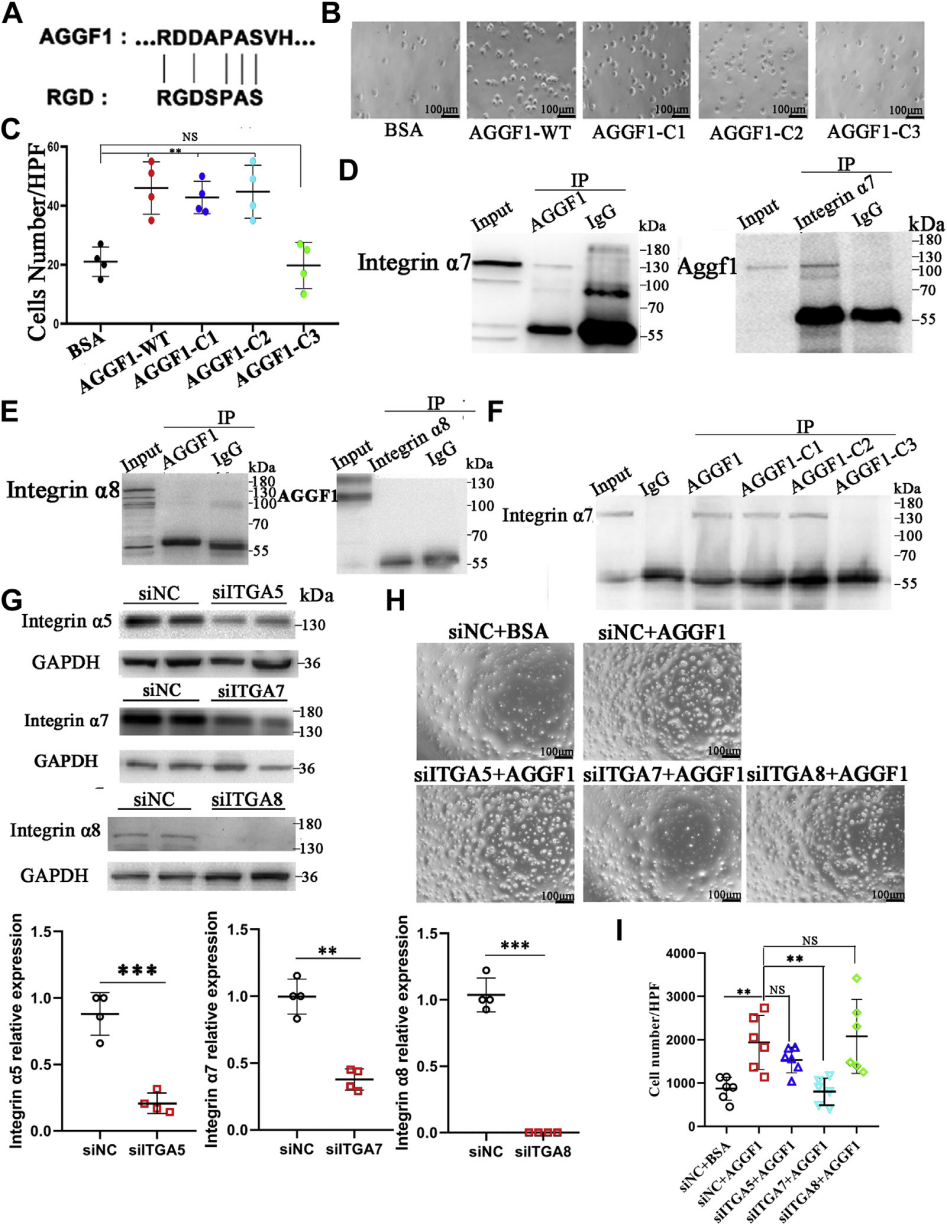
**Identification of integrinα7 as a candidate receptor for AGGF1.***A*, sequence alignment of an AGGF1 RDD domain and the integrin RGD domain from fibronectin. *B* and *C*, cell adhesion assays. MOVAS-1 cells were plated in wells coated with 20 μl control BSA or 20 μl of WT AGGF1, AGGF1-C1, AGGF-C2 or AGGF1-C3 protein (5 μg/ml). After 1 h, unbound cells were washed away, and bound cells were photographed and analyzed (mean ± SD, one-way ANOVA with Dunnett test for multiple comparison; ∗∗*p* < 0.01, ∗∗∗*p* < 0.001, n = 4/group). *D*, Co-IP analysis showing interaction between AGGF1 and integrin α7 (ITGA7). HeLa cells were cotransfected with *AGGF1* and *ITGA7* expression plasmids for 48 h, lysed, and used for immunoprecipitation. *Left panel*, Co-IP with anti-AGGF1 for immunoprecipitation and anti-integrin α7 for Western blotting. *Right panel*, reciprocal Co-IP with anti-integrin α7 for immunoprecipitation and anti-AGGF1 for Western blotting. *E*, Co-IP analysis showing that AGGF1 does not interact with integrin α8 (ITGA8). HeLa cells were cotransfected with *AGGF1* and *ITGA8* expression plasmids for 48 h, lysed, and used for immunoprecipitation. *Left panel*, Co-IP with anti-AGGF1 for immunoprecipitation and anti-integrin α8 for Western blotting. *Right panel*, reciprocal Co-IP with anti-integrin α8 for immunoprecipitation and anti-AGGF1 for Western blotting. *F*, Co-IP analysis showing the interaction domain between AGGF1 and integrin α7 is between AGGF1-C2 AGGF1-C3. WT AGGF1, AGGF1-C1 and AGGF1-C2 shows interaction with integrin α7, but the effect was lost with AGGF1-C3. HeLa cells were cotransfected with *ITGA7* and wild-type or mutant *AGGF1* expression plasmids (AGGF1-C1, AGGF1-C2 or AGGF1-C3) for 48 h, lysed, and used for immunoprecipitation. Anti-AGGF1 was used for immunoprecipitation and anti-integrin α7 was used for Western blotting. *G*, Western blot analysis showing successful knockdown of integrin α5, α7 and α8 in MOVAS cells. MOVAS cells were transfected with siRNA for *ITGA5* (siITGA5), *ITGA7* (siITGA7), *ITGA8* (siITGA8), or control siNC for 48 h, lysed, and used for Western blotting. Data are shown as mean ± SD (Student’s *t* test; ∗∗*p* < 0.01, ∗∗∗*p* < 0.001, n = 4/group). *H* and *I*, cell adhesions assays. MOVAS cells were transfected with siRNA for *ITGA5* (siITGA5), *ITGA7* (siITGA7), *ITGA8* (siITGA8), or control siNC and plated in wells coated with 20 μl of AGGF1 (5 μg/ml) for 1 h. Unbound cells were washed away, and bound cells were photographed (*H*) and analyzed (*I*) (mean ± SD, one-way ANOVA with Dunnett test for multiple comparison; ∗∗*p* < 0.01, n = 6/group). NS, not significant. AGGF1, angiogenic factor with G patch and FHA domains 1.

Careful examination of amino acid sequences between 574 and 614 revealed an interesting RDD domain (RDDAPAS), which has sequence homology to the RGD domain of fibronectin (RGDSPAS) (Fig. 3*A*). Fibronectin was shown to be involved in integrin binding and cell adhesion (13). Therefore, we hypothesized that the critical AGGF1 domain involved in VSMC phenotypic switching is involved in adhesion of AGGF1 to VSMCs *via* an integrin. To test the hypothesis, we performed MOVAS-1 cell adhesion assays (Fig. 3, *B* and *C*). MOVAS-1 cells showed strong adhesion to wells coated with purified AGGF1-WT, AGGF1-C1, and AGGF1-C2, but not with AGGF1-C3 (Fig. 3, *B* and *C*). These results suggest that the VSMC phenotypic switching domain of AGGF1 between amino acids 564 and 614 is also involved in the interaction between AGGF1 and MOVAS-1 cells.

Similar to AGGF1, integrin α7 (ITGA7) and α8 (ITGA8) were reported to be involved in vascular injury and promoting the development of the contractile phenotype of VSMCs (14, 15). Thus, we assessed whether AGGF1 interacted with integrin α7 and integrin α8. Co-IP analysis showed that AGGF1 successfully precipitated integrin α7 (Fig. 3*D*), but failed to precipitate integrin α8 (Fig. 3*E*). Further Co-IP analysis showed that AGGF1-C3 failed to interact with ITGA7, while AGGF1-C1 and AGGF1-C2 showed robust interaction with integrin α7 as WT AGGF1 (Fig. 3*F*). The data suggest that the AGGF1 interacts with integrin α7 through a domain between AGGF1-C2 and AGGF1-C3 (*i.e.*, the phenotypic switching domain between amino acids 564 and 614).

We recently found that AGGF1 regulated endothelial cell (EC) functions and angiogenesis by interacting with its receptor integrin α5 on endothelial cell surface (16). As integrin α5 is also expressed in VSMCs, we investigated whether integrin α5 mediated the adhesion of AGGF1 to VSMCs. We used siRNAs to knock the expression of *ITGA5*, *ITGA7*, and *ITGA8* down (Fig. 3*G*) and then performed cell adhesions assays for MOVAS-1 cells treated with AGGF1 or control BSA. As shown in Figure 3, *H* and *I*, MOVAS-1 cells showed strong adhesion to wells coated with AGGF1 (compare AGGF1 with BSA in the siNC groups); however, the effect was inhibited by siITGA7, but not by siITGA5 and siITGA8. The data suggest that integrin α7, but not α5 or α8, is involved in the interaction between AGGF1 and MOVAS-1 cells.

**Figure 4:**
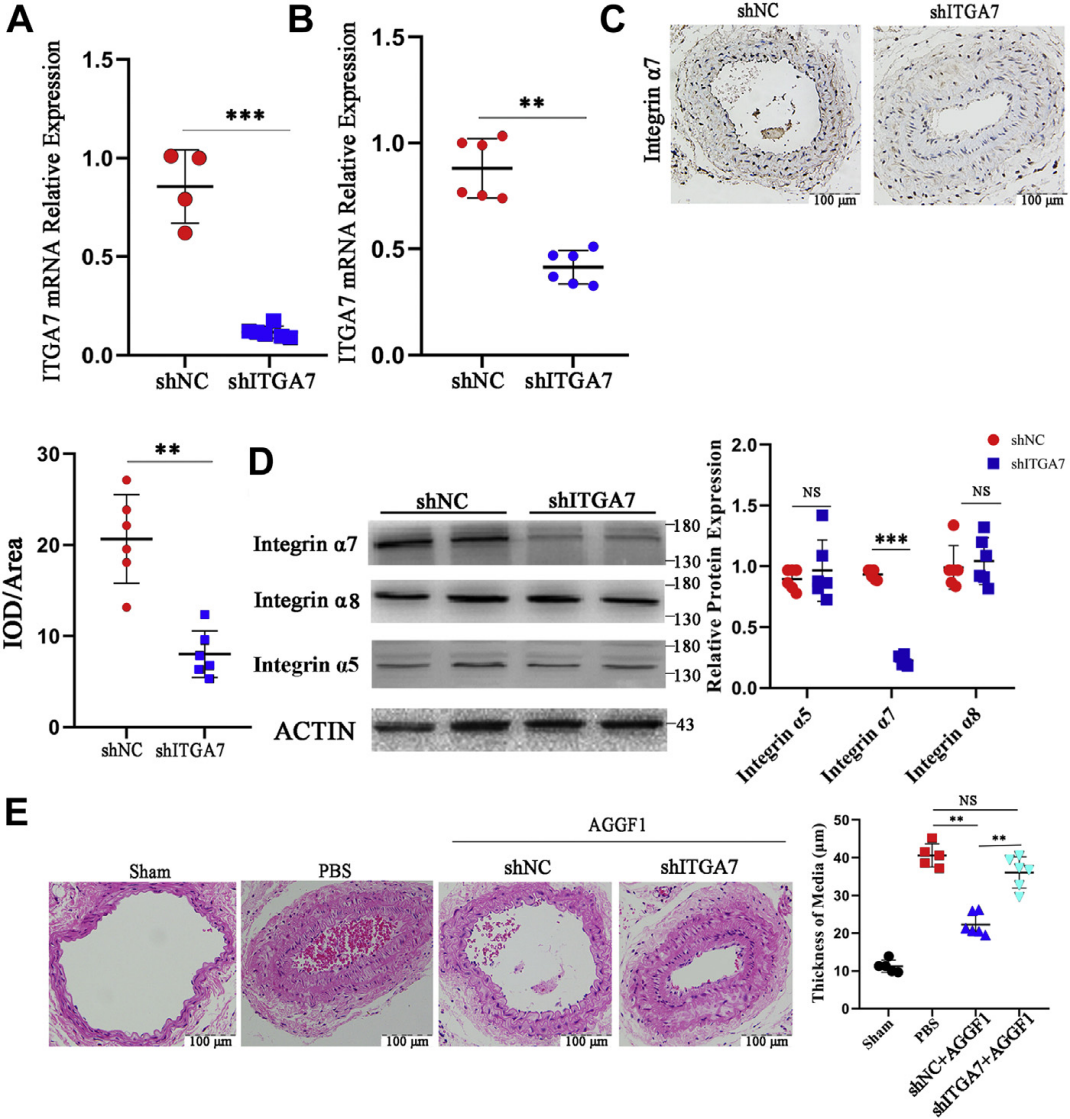
**Knockdown of *ITGA7* expression reverses the inhibitory effect of AGGF1 on intimal hyperplasia after vascular injury in mice**. *A* and *B*, quantitative RT-PCR analysis showed that compared with control shNC, shRNA for *ITGA7* successfully knocked expression of *ITGA7* down in MOVAS cells 96 h after lentivirus infection (*A*) and in mouse vascular tissues 6 weeks after the tail vein injection of lentiviruses (*B*). ∗∗*p* < 0.01, ∗∗∗*p* < 0.001, n = 4 to 6/group (Student’s *t* test). *C*, immunostaining showed that compared with control shNC, shRNA for *ITGA7* successfully knocked expression of integrin α7 down in blood vessels in mice. ∗∗*p* < 0.01, n = 6/group (Student’s *t* test). *D*, Western blot analysis showed successful knockdown of integrin α7, but not α8 or α5, by shRNA for *ITGA7* as compared with shNC in mouse vascular tissue samples 6 weeks after lentivirus injection. ∗∗∗*p* < 0.001, n = 6/group (Student’s *t* test). *E*, H&E staining showed that AGGF1 protein treatment blocked neointimal formation after vascular injury (shNC+AGGF1) as compared with PBS, however, the effect was inhibited by shRNA for *ITGA7* (shITGA7+AGGF1). ∗∗*p* < 0.01, n = 6/group (Student’s *t* test). NS, not significant. AGGF1, angiogenic factor with G patch and FHA domains 1.

We developed a lentiviral shRNA system (shITGA7 *versus* control shNC) to knock the expression of *ITGA7* down in mice. Quantitative RT-PCR showed that compared with shNC, shITGA7 successfully reduced *ITGA7* expression in both VSMCs (Fig. 4*A*) and mouse vascular tissues (Fig. 4*B*). Western blot analysis and immunostaining analysis showed that the protein level of integrin α7, but not that of integrin α5 and α8, in vascular tissues was significantly reduced after injection of shITGA7 lentivirus (Fig. 4, *C* and *D*). In mice with wire-induced vascular injury, AGGF1 protein therapy for 28 days blocked neointima formation; however, the effect was reversed by shITGA7 (Fig. 4*E*). The results indicate that AGGF1 protein blocks neointima formation after vascular injury through integrin α7 *in vivo*.

**Figure 5:**
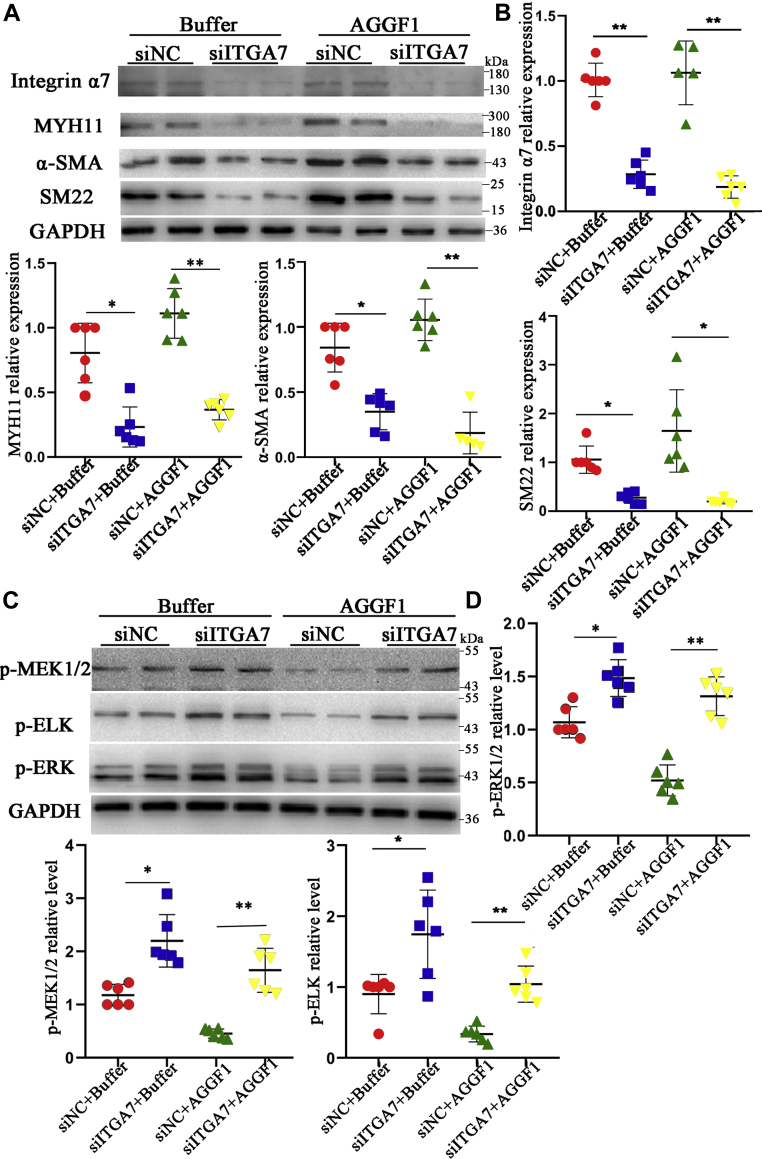
**AGGF1 upregulates phenotypic switching of VSMCs via *ITGA7*.***A*, Western blot analysis showed that AGGF1 enhanced the expression of MYH11, α-SMA, and SM22; however, the effect was inhibited by knockdown of *ITGA7* with siITGA7. MOVAS cells were transfected with control siNC or *ITGA7* siRNA (siITGA7), incubated with 20 μl control PBS or 20 μl of AGGF1 (5 μg/ml) for 24 h, lysed, and used for Western blot analysis. *B*, quantification of Western blotting images as in (*A*) (mean ± SD, one-way ANOVA with Dunnett test for multiple comparison; ∗*p* < 0.05, ∗∗*p* < 0.01, n = 5–6/group). *C*, Western blotting showed that AGGF1 inhibited phosphorylation of MEK1/2 and ERK1/2, but the effect was reversed by knockdown of *ITGA7* with siITGA7. MOVAS cells were treated as in (*A*). *D*, quantification of Western blotting images as in (*C*) (mean ± SD, one-way ANOVA with Dunnett test for multiple comparison; ∗∗*p* < 0.01, n = 6/group). AGGF1, angiogenic factor with G patch and FHA domains 1; ERK1/2, extracellular signal-regulated kinases ½; MEK1/2, MAPK/ERK kinase ½.

Our previous study suggested that AGGF1 blocked neointimal formation after vascular injury and VSMC phenotypic switching by regulating the MEK-ERK-ELK signaling pathway (6). Thus, we assessed whether *ITGA7* is required for AGGF1 signaling in VSMCs. MOVAS-1 cells were transfected with siITGA5、siITGA7, or siITGA8 (siNC as negative control), treated with or without AGGF1, and used for Western blot analysis. siITGA5, siITGA7, or siITGA8 successfully reduced the expression of integrin α5, α7 and α8 by >95%, 70%, and >80%, respectively (Figs. 5*A* and S3, *A* and *B*). Consistent with the earlier reports (14, 15), knockdown of integrins α7 or α8 significantly reduced the expression of smooth muscle contractile markers MYH11, α-SMA, and SM22 (Figs. 5, *A* and *B* and S3*A*) and increased activation of MEK1/2, ERK1/2, and ELK (Figs. 5, *C* and *D* and S4*A*). On the other hand, knockdown of integrin α5 significantly reduced the expression of smooth muscle contractile markers (Fig. S3*B*), but decreased the phosphorylation of MEK1/2, ERK1/2, and ELK (Fig. S4*B*). AGGF1 treatment significantly increased the expression of smooth muscle contractile markers (Fig. 5, *A* and *B*) and inhibited activation of MEK1/2, ERK1/2 and ELK; however, the effects were inhibited by siITGA7 (Fig. 5, *C* and *D*). The data suggest that AGGF1 regulates VSMC functions through integrin α7. AGGF1 treatment significantly increased the expression of smooth muscle contractile markers and inhibited activation of MEK1/2, ERK1/2, and ELK (Figs. S3*A* and S4*A*). These effects were not affected by siITGA8 or siITGA5 (Figs. S3*A* and S4*A*), suggesting that AGGF1 regulates VSMC functions independent of integrin α8 or α5.

### Only the first arginine residue of the RDD motif is essential for interaction between AGGF1 and integrin α7

**Figure 6:**
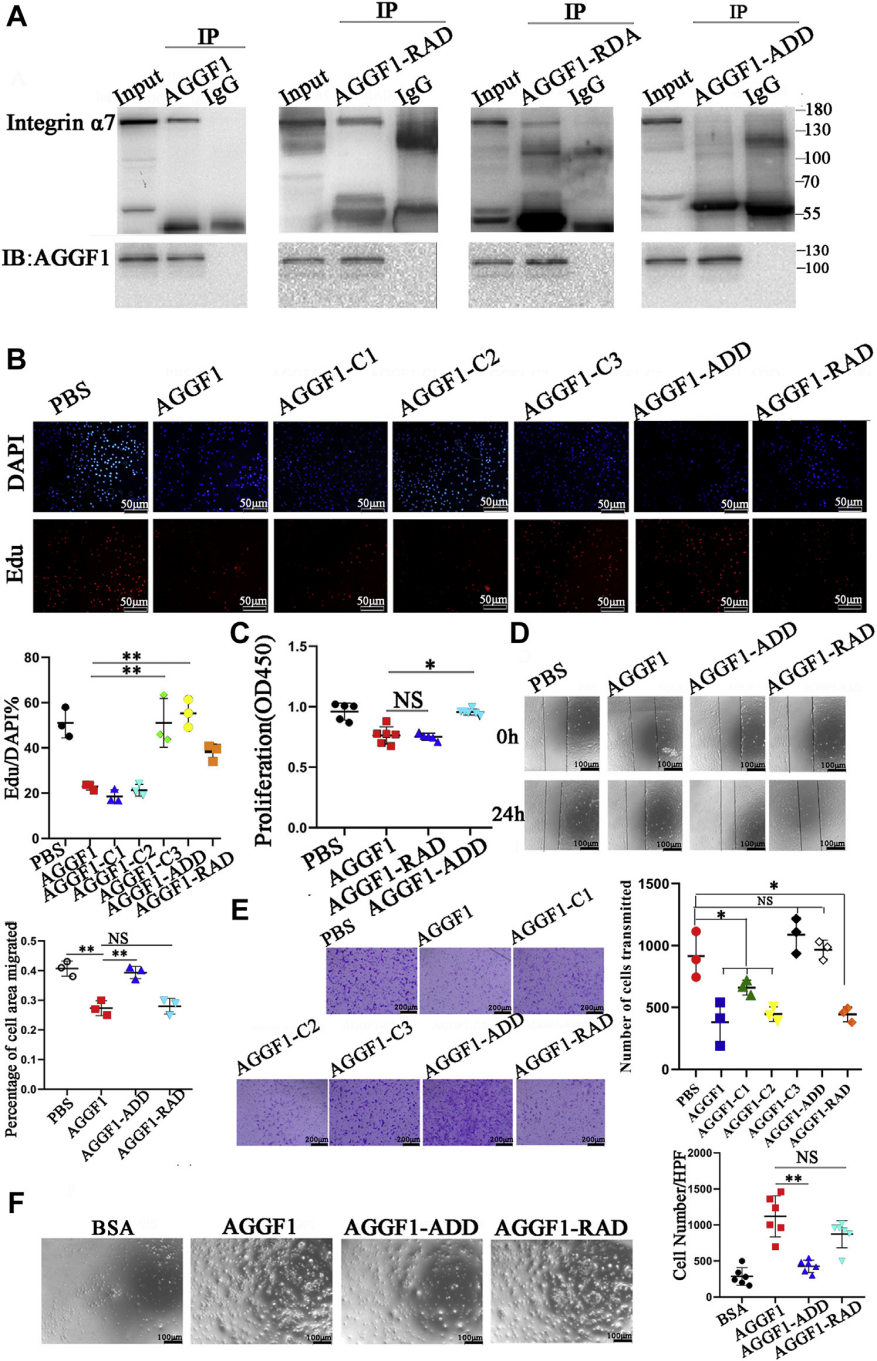
**The arginine residue of the RDD motif is critical to the interaction between AGGF1 and integrin α7.***A*, Co-IP analysis showed that mutant AGGF1-ADD failed to interact with integrin α7, however, AGGF1-RAD and AGGF1-RDA continued to interact with integrin α7. HeLa cells were co-transfected with *ITGA7* and wild-type or mutant *AGGF1* expression plasmids (AGGF1-RAD, AGGF1-RDA, AGGF1-ADD) for 48 h, lysed, and used for immunoprecipitation. Anti-AGGF1 was used for immunoprecipitation and anti-integrin α7 was used for Western blotting. *B*, cell proliferation analysis with EdU staining showed that AGGF1-WT, AGGF1-RAD, AGGF1-C1, and AGGF1-C2 inhibited the proliferation of VSMCs, but AGGF1-C3 and AGGF1-ADD lost this effect. MOVAS cells were treated with 20 μl control PBS or 20 μl of WT AGGF1 or mutant AGGF1 (AGGF1-C1, AGGF1-C2, AGGF1-C3, AGGF1-ADD, and AGGF1-RAD) (5 μg/ml), cultured to 90% density, and incubated with the EdU dye solution for 2 h. Cells were fixed and photographed. The upper DAPI shows the nucleus staining, and the lower EDU staining shows the newly proliferated cells. The number of EdU positive, fluorescent cells in all images was calculated using Image Pro Plus 6.0 and analyzed (mean ± SD, one-way ANOVA with Dunnett test for multiple comparison; ∗∗*p* < 0.01, n = 3/group). *C*, CCK8 cell proliferation analysis showed that AGGF1, AGGF1-RAD inhibited the proliferation of VSMCs, but AGGF1-ADD lost this effect. MOVAS cells were treated as in (*B*) but for 36 h and analyzed with the CCK8 solution (mean ± SD, one-way ANOVA with Dunnett test for multiple comparison; ∗*p* < 0.05, ∗∗*p* < 0.01, n = 6/group). *D*, the AGGF1 and mutant AGGF1-RAD protein inhibited the migration of smooth muscle cells, but the AGGF1-ADD mutant protein lost this effect in scratch-wound healing assays. MOVAS cells were cultured in a 6-well plate overnight, and a scratch was made on the bottom of wells. A total of 20 μl of wild type AGGF1 (AGGF1-WT) (5 μg/ml), mutant AGGF1 (AGGF1-RAD, AGGF1-ADD) (5 μg/ml), or 20 μl PBS control was added. The cells were allowed to migrate for 24 h. The degree of cell migration was quantified and shown (mean ± SD, one-way ANOVA with Dunnett test for multiple comparison; ∗∗*p* < 0.01, n = 3/group). *E*, cell migration analysis of MOVAS cells in Boyden chambers for 12 h treated with WT or mutant AGGF1. MOVAS cells were plated in Boyden chambers, and treated with 20 μl of wild-type AGGF1 (AGGF1-WT) (5 μg/ml), mutant AGGF1 (AGGF1-C1, AGGF1-C2, AGGF1-C3, AGGF1-RAD, AGGF1-ADD) (5 μg/ml), or 20 μl PBS control. 500 μl of serum-containing culture medium was added to the bottom of the chamber. After 12 h, cells above the chamber were wiped off, and cells at the bottom of the chamber were fixed, stained with crystal violet, photographed, and analyzed (mean ± SD, one-way ANOVA with Dunnett test for multiple comparison; ∗∗*p* < 0.01, n = 6/group). *F*, cell adhesion assays. MOVAS cells were plated in wells coated with 20 μl control PBS or 20 μl of WT AGGF1, AGGF1-RAD, and AGGF1-ADD (5 μg/ml). After 1 h, unbound cells were washed away, and bound cells were photographed and analyzed (mean ± SD, one-way ANOVA with Dunnett test for multiple comparison; ∗*p* < 0.05, n = 6/group). NS, not significant. AGGF1, angiogenic factor with G patch and FHA domains 1.

**Figure 7:**
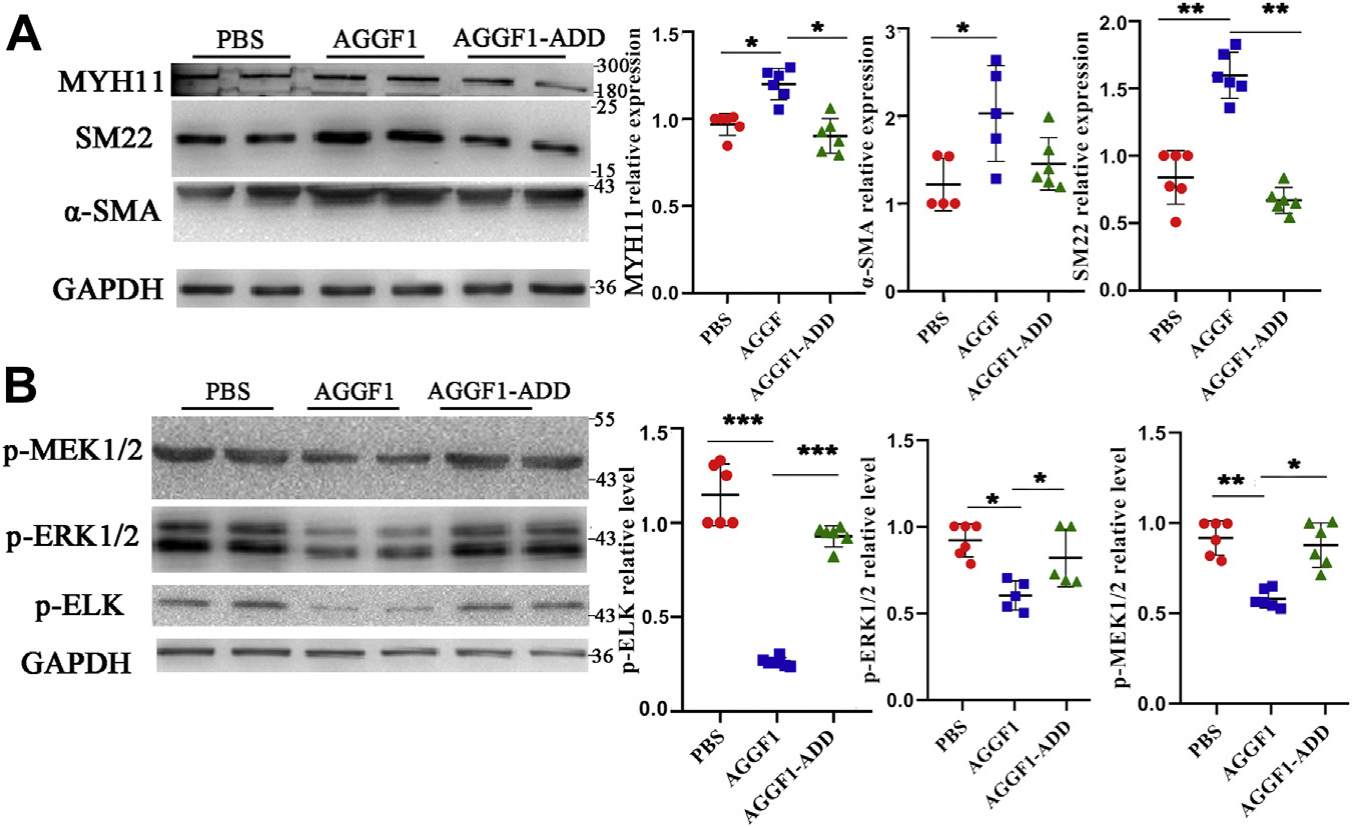
**Effect of AGGF1-ADD mutant protein on smooth muscle cells.***A*, Western blot analysis showed that AGGF1 enhanced expression of MYH11, α-SMA, and SM22, but the effect was inhabited by the AGGF1-ADD mutation. MOVAS cells were treated with 20 μl control PBS or 20 μl of wild type AGGF1 or mutant AGGF1 AGGF1-ADD (5 μg/ml) for 24 h, lysed, and used for Western blot analysis (mean ± SD, one-way ANOVA with Dunnett test for multiple comparison; ∗*p* < 0.05, ∗∗*p* < 0.01, n = 6/group). *B*, Western blot analysis showed that AGGF1 inhibited phosphorylation of ELK, MEK1/2, and ERK1/2, but the effect was reversed by the AGGF1-ADD mutation. MOVAS cells were treated as in (*A*) but for 15 min, lysed and used for Western blot analysis (mean ± SD, one-way ANOVA with Dunnett test for multiple comparison; ∗*p* < 0.05, ∗∗*p* < 0.01, ∗∗∗*p* < 0.001, n = 6/group). AGGF1, angiogenic factor with G patch and FHA domains 1; ERK1/2, extracellular signal-regulated kinases ½; MEK1/2, MAPK/ERK kinase ½.

To identify the key amino acid that is required for interaction between AGGF1 and integrin α7, we mutated each residue of the RDD motif to an alanine residue, resulting in three mutant AGGF1 proteins AGGF1-ADD, AGGF1-RAD, and AGGF1-RDA. Through Co-IP analysis, we found that compared with wild-type AGGF1, only mutant AGGF1-ADD lost its function for interaction with integrin α7, while AGGF1-RAD and AGGF1-RDA mutants retained the interaction with integrin α7 (Fig. 6*A*). The data indicate that only the first arginine residue of the RDD motif is required for interaction between AGGF1 and integrin α7. Functionally, similar to AGGF1-C3, mutant AGGF1-ADD mutant, but not mutant AGGF1-RAD, lost the effects of AGGF1 on inhibition of smooth muscle cell proliferation (Fig. 6, *B* and *C*), migration in both cell scratch-wound healing assays and Boyden-chamber Transwell assays (Fig. 6, *D* and *E*), and adhesion (Fig. 6*F*) compared with WT AGGF1, AGGF1-C1, and AGGF-C2. Moreover, compared with WT AGGF1, mutant AGGF1-ADD lost the effects of AGGF1 on increasing the levels of intracellular contractile markers MYH11, SM22, and α-SMA (Fig. 7*A*) and inhibiting phosphorylation of MEK1/2, ELK, and ERK1/2 in VSMCs (Fig. 7*B*). On the contrary, these functions were retained by mutant AGGF1-RAD (Fig. S5. *A* and *B*). Together, the data indicate that only the first arginine residue of the RDD motif is required for interaction between AGGF1 and integrin α7 and critical for the function of AGGF1 in VSMCs.

### The phenotypic switching domain/AGGF1-integrin α7 interaction domain is involved in blocking neointimal formation after vascular injury in mice

**Figure 8:**
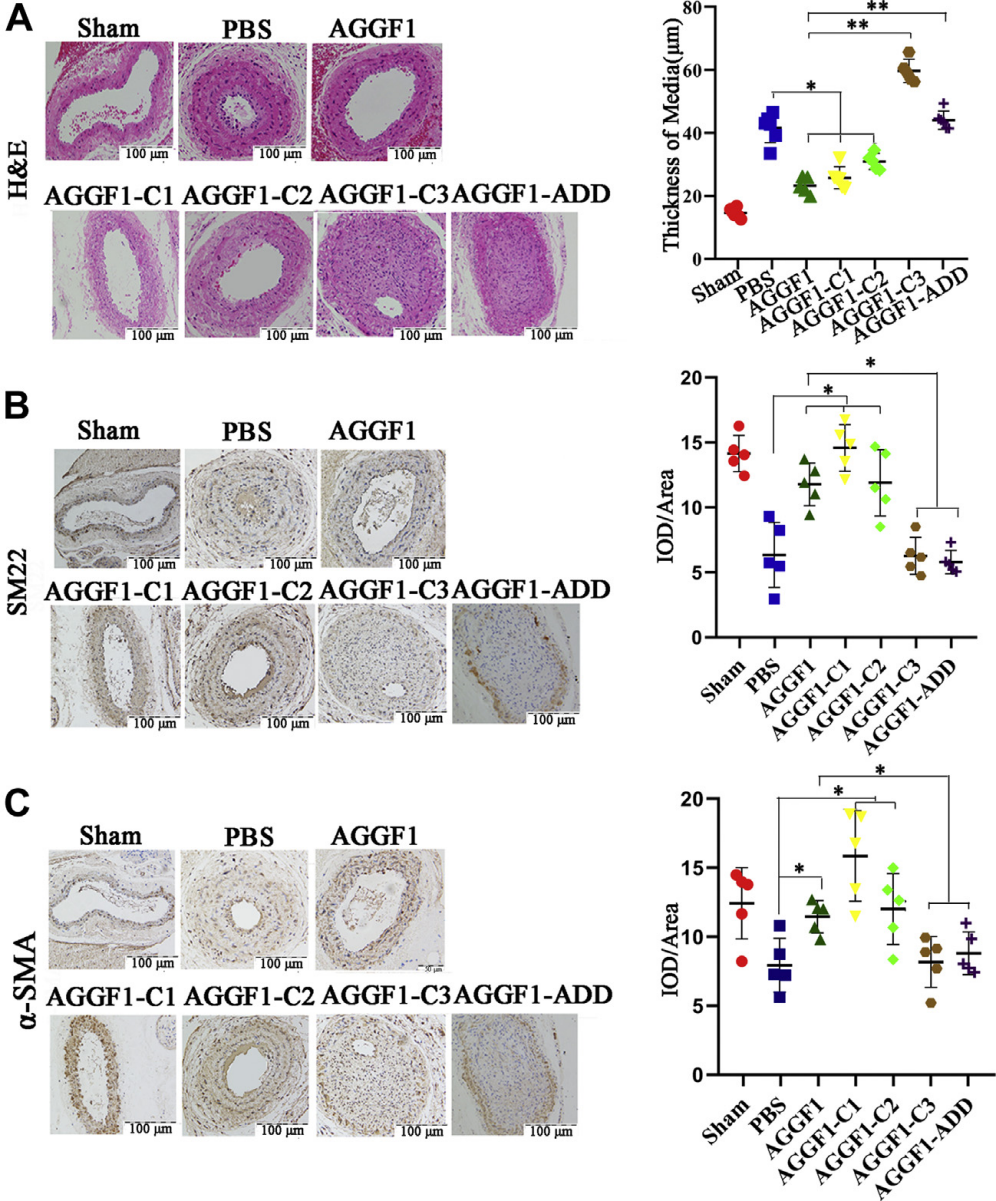
**Analyses of AGGF1-WT, AGGF1-C1 and AGGF1-C2, AGGF1-C3 and AGGF1-ADD on *in vivo* neointimal formation and VSMC proliferation after vascular injury in mice.** The left carotid artery of 10- to 12-week-old C57BL6 mice was damaged by a guide wire, and wild-type or mutant AGGF1 (100 μl × 1.2 μM) was intraperitoneally injected once every 3 days. The mice were sacrificed after 4 weeks, and the vascular tissues were collected for H&E staining (*A*), and immunostaining for SM22 (*B*) or α-SMA (*C*). *A*, AGGF1-WT, AGGF1-C1, and AGGF1-C2 decreased neointimal formation after vascular injury in mice, but AGGF1-C3 and AGGF1-ADD lost the effect. Sham, the isolated right carotid artery without guide wire damage. *B* and *C*, AGGF1-WT, AGGF1-C1 and AGGF1-C2 can maintain the function of smooth muscle cells after vascular injury as shown by immunostaining for SM22 (*B*) and α-SMA (*C*); however, AGGF1-C3 and AGGF1-ADD lost the function. The data are shown as mean ± SD (one-way ANOVA with Dunnett test for multiple comparison; ∗*p* < 0.05, ∗∗*p* < 0.01, n = 6/group). PBS was used as a negative control. AGGF1, angiogenic factor with G patch and FHA domains 1; VSMCs, vascular smooth muscle cells.

In order to examine whether the interaction between AGGF1 and integrin α7 plays an important role *in vivo*, we injected AGGF1-WT, AGGF1-C1, AGGF1-C2, AGGF1-C3, or AGGF1-ADD (0.12 nmole) into mice with wire-induced vascular injury in the carotid arteries by i.p. to determine their effects on neointimal formation. Prior to the study, we measured the stability of purified wild-type and mutant AGGF1 at 37 ^°^C (Fig. S6). The half-life of wild-type AGGF1 (52.19 h) and mutant AGGF1 used for mouse studies was similar (66.16 h, 57.22 h, 61.61 h, and 58.29 h for AGGF1-C1, AGGF1-C2, AGGF1-C3, and AGGF1-ADD, respectively) (Fig. S6*C*). The same mole of wild-type or mutant AGGF1 was injected into mice with wire-induced vascular injury once every 3 days (72 h). Wild-type AGGF1 protein therapy effectively inhibited neointimal formation after vascular injury compared with PBS control without any differences between male mice and female mice (Figs. 8*A* and S7). The treatments of mice with AGGF1-WT, AGGF1-C1, and AGGF1-C2 (all containing the AGGF1-α7 interaction domain) effectively inhibited neointimal formation after vascular injury compared with PBS control; however, the effect got lost for AGGF1-C3 without the AGGF1-α7 interaction domain or mutant AGGF1-ADD with the RDD motif mutated into ADD motif (Fig. 8*A*). Similarly, immunostaining analysis showed that AGGF1-WT, AGGF1-C1, and AGGF1-C2 significantly increased the expression of α-SMA and SM22 in carotid arteries compared with PBS control; however, the effect got lost for AGGF1-C3 and AGGF1-ADD (Fig. 8, *B* and *C*).

**Figure 9:**
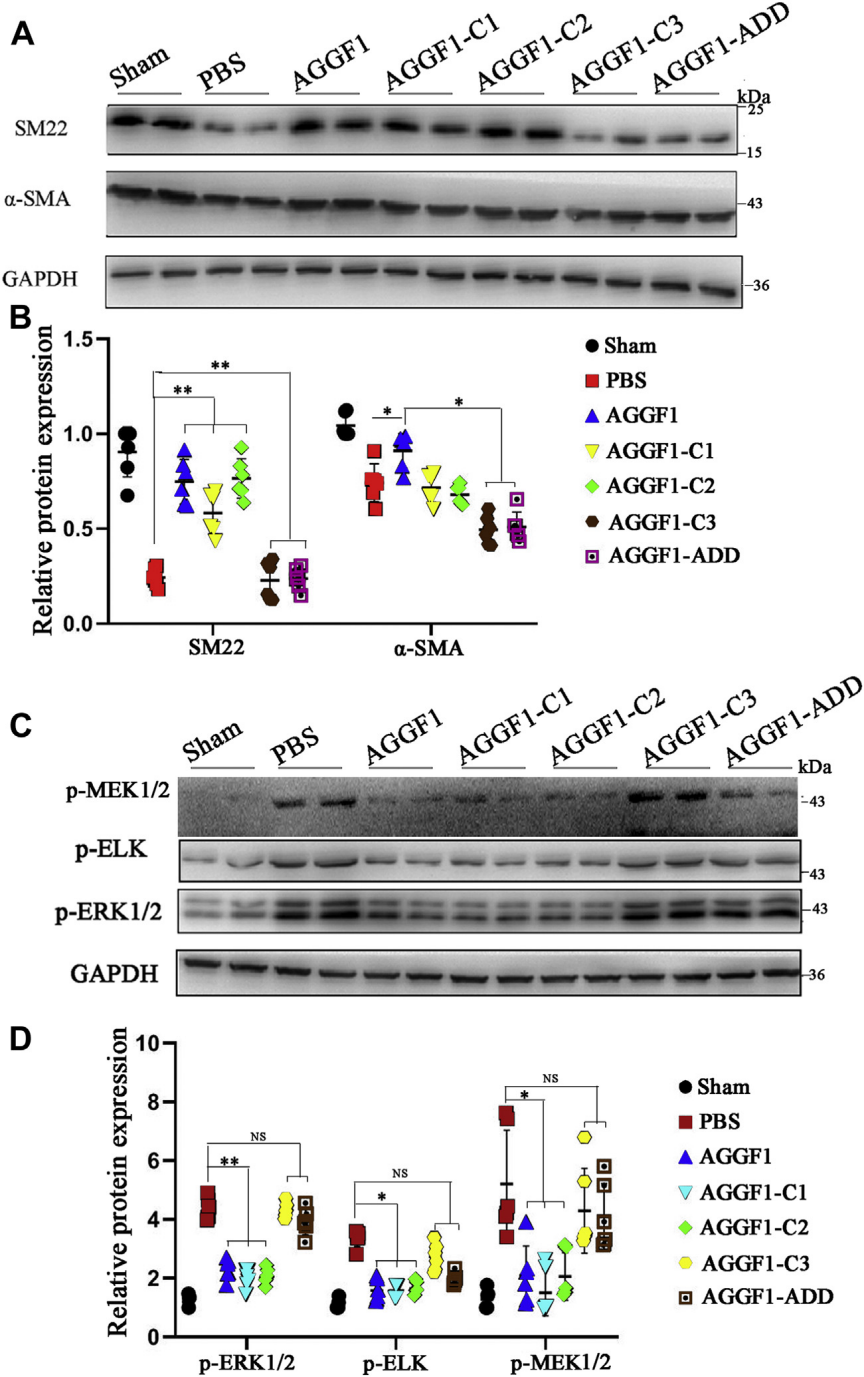
***In vivo* analyses of AGGF1-WT, AGGF1-C1 and AGGF1-C2, AGGF1-C3 and AGGF1-ADD on expression of VSMC phenotypic switching markers and MEK/ERK1/2/ELK signaling.***A* and *B*, Western blot analysis of carotid artery samples after vascular injury shows that AGGF1-WT, AGGF1-C1, and AGGF1-C2 increased the expression of α-SMA and SM22, but AGGF1-C3 and AGGF1-ADD lost the effect. After the left carotid artery of 10- to 12-week-old C57BL6 mice was damaged by a guide wire, wild-type AGGF1 or mutant AGGF1 (AGGF1-C1, AGGF1-C2, AGGF1-C3, and AGGF1-ADD) (100 μl × 1.2 μM) was intraperitoneally injected every 3 days. After 4 weeks, the mice were sacrificed, and the vascular tissue was collected, homogenized, and used to for Western blot analysis. Sham, the isolated right carotid artery without guide wire damage. *C* and *D*, Western blot analysis of carotid artery samples after vascular injury shows that the phosphorylation of MEK1/2, ERK1/2, and ELK was decreased with treatment of AGGF1-WT, AGGF1-C1 and AGGF1-C2, but not of AGGF1-C3 and AGGF1-ADD. PBS was used as a negative control. The data are shown as mean±SD (one-way ANOVA with Dunnett test for multiple comparison; ∗*p* < 0.05, ∗∗*p* < 0.01, n = 5–6/group). AGGF1, angiogenic factor with G patch and FHA domains 1; ERK1/2, extracellular signal-regulated kinases ½; MEK1/2, MAPK/ERK kinase ½.

In addition, we also examined the expression of VSMC phenotypic switching markers and MEK1/2, ERK1/2, and ELK signal using Western blot analysis with dissected carotid artery samples from mice. AGGF1-WT, AGGF1-C1, and AGGF1-C2 significantly increased the expression of α-SMA and SM22 compared with PBS control; however, the effect got lost for AGGF1-C3 and AGGF1-ADD (Fig. 9, *A* and *B*). The phosphorylation of MEK1/2, ERK1/2, and ELK was decreased for treatments with AGGF1-WT, AGGF1-C1, and AGGF1-C2, but not with AGGF1-C3 and AGGF1-ADD (Fig. 9, *C* and *D*). Together, these data show that the RDD interaction domain between AGGF1 and integrin α7 (amino acids 564–614) is critical to AGGF1-driven inhibition of neointimal formation after vascular injury by promoting VSMC phenotypic switching to a contractile state and by inhibiting MEK1/2, ERK1/2, and ELK signaling.

## Discussion

Neointimal formation causes restenosis after vascular injury during the clinical treatment of CAD using PCI (3, 17, 18, 19, 20). Our previous study showed that systemic delivery of purified AGGF1 protein blocked neointimal formation after vascular injury in a mouse model (6). We also showed that in VSMCs, AGGF1 inhibited PDGF-induced phosphorylation of MEK, ERK1/2, and ELK, and dephosphorylated ELK failed to displace myocardin from the SRF–CArG complex, resulting in increased expression of contractile markers MYH11, α-SMA, and SM22 (6). Increased expression of contractile markers led to the phenotypic switching of VSMCs to the contractile phenotype with a less potential of proliferation and migration, thereby blocking neointimal formation and restenosis (6). However, it is unknown how AGGF1 inhibits phosphorylation of MEK, ERK1/2, and ELK and a follow-up cascade of signaling events. The key finding from this study is that the functions of AGGF1 in VSMCs are dependent on integrin α7. Multiple lines of evidence suggest that integrin α7 is the cell surface receptor for AGGF1 on VSMCs. First, integrins are well-known cell surface receptors for many ligands (21), and our Co-IP analysis demonstrated the interaction between AGGF1 and integrin α7 (Fig. 3, *A*–*D*). The specificity of the interaction between AGGF1 and integrin α7 was shown by the lack of interaction between AGGF1 and integrin α8, another major integrin in VSMCs (Fig. 3*E*). Second, the domain for the interaction between AGGF1 and integrin α7 was mapped to a 50 amino acid region (amino acids 564–614) by the finding that AGGF1-C2 interacts with integrin α7, but the AGGF1-C3 failed to do so (Figs. 1 and 3*F*). The deletion of the 50 amino acid AGGF1-α7 interaction domain from 564 to 614 abolished the function of AGGF1 in enhancing expression of contractile markers in MOVAS-1 cells (Figs. S2 and 1) and in mouse carotid arteries after vascular injury (Fig. 9, *A* and *B*). Moreover, the deletion of the AGGF1-α7 interaction domain reversed the function of AGGF1 in inhibiting MEK-ERK1/2-ELK signaling in mouse carotid arteries after vascular injury (Fig. 9, *C* and *D*). Third, knockdown of expression of *ITGA7*, but not *ITGA8*, reversed the function of AGGF1 in inhibiting MEK-ERK1/2-ELK signaling (Fig. 5). The inhibitory effect of AGGF1 on neointimal formation after vascular injury was blocked by shRNA for *ITGA7* in mice (Fig. 4). Fourth, the deletion of the AGGF1-α7 interaction domain reversed the functions of AGGF1 in inhibiting MOVAS-1 proliferation and migration, and regulation of key genes involved in cell cycle regulation and proliferation (Fig. 4), and cell proliferation and neointimal formation in mouse carotid arteries after vascular injury (Fig. 2). These results suggest that AGGF1 blocks neointimal formation after vascular injury by interacting with integrin α7 on the surface of VSMCs and regulating a cascade of signaling events involved in MEK-ERK1/2-ELK signaling, phenotypic switching, cell proliferation, cell cycle regulation, and cell migration. Our study provides important insights into a novel AGGF1-based therapy for blocking neointimal formation and restenosis after treatments of vascular diseases.

Our studies with a series of studies with N-serial deletions and C-serial deletions of AGGF1 indicate that the critical AGGF1 domain involved in VSMCs phenotypic switching, proliferation, and migration, cell cycle regulation, and regulation of key cell cycle and proliferation genes is located between amino acids 574 and 614 (Fig. S2 and 2). The same domain is also involved in the interaction between AGGF1 and MOVAS-1 cells (Fig. 3). Careful examination of amino acid sequences between 574 and 614 revealed an interesting RDD domain (RDDAPAS), which has sequence homology to the RGD domain of fibronectin (RGDSPAS) (Fig. 3*A*). Alanine-scanning mutagenesis of the RDD motif, Co-IP analysis, and other functional studies *in vitro* and *in vivo* showed that only the first arginine residue of the RDD motif is required for interaction between AGGF1 and integrin α7 and for the function of AGGF1 in VSMCs and in neointima formation after vascular injury in mice (Figure 5, Figure 6, Figure 7, Figure 8). However, caution is needed for interpretation of the data. First, mutations of each of the three RDD residues into alanine may be too weak to disrupt the interaction between AGGF1 and integrin α7 and alter the functions of AGGF1. Future studies with more severe mutations such as mutations of D to positively charged R/K or others structurally different amino acids may address this issue. Moreover, the interaction between AGGF1 and integrin α7 may require other amino acid residues outside of RDD, and future studies with mutations of amino acids spanning RDD may address this issue.

The data from this study suggest that the AGGF1-integrin α7 pathway is a major signaling pathway that regulates the functions of VSMCs and modulates neointimal formation and restenosis after vascular injury. This conclusion is further supported by earlier studies on integrin α7. It is interesting that the expression of integrin α7 was increased in a model of atherosclerosis in rats and by PDGF in VSMCs (22). Global *Itga7*^*−/−*^ KO mice deficient in integrin α7 showed VSMCs abnormalities, including hyperplasia and hypertrophy (23). VSMC-specific *Itga7* KO mice showed significantly reduced expression of VSMC contractile proteins in response to vascular injury and increased neointimal formation and reduced vascular compliance (15). Similarly, we showed that heterozygous *Aggf1*^*+/−*^ KO mice exhibited significantly increased neointimal formation and significantly reduced expression of VSMC contractile proteins after vascular injury (6). These data indicate that integrin α7 and AGGF1 share similar functions in the biology of VSMCs, further supporting our finding that integrin α7 is a receptor for AGGF1 in VSMCs.

Many different types of integrins were reported in VSMCs, including α1β1, α2β1, α3β1, α4β1, α5β1, α6β1, α7β1, α8β1, αvβ1, αvβ3, αvβ5, and α6β4 (24). However, only integrin α7 and integrin α8 were reported to promote the contractile phenotype of VSMCs (14, 15). Our data excluded integrin α8 as a potential receptor for AGGF1 (Fig. 3). Integrin α8 did not interact with AGGF1 (Fig. 3). Knockdown of *ITGA8* expression did not affect the adhesion of VSMCs to AGGF1 (Fig. 3) nor the effects of AGGF1 on expression of contractile proteins a-SMA and SM22 or phosphorylation of MEK, ERK1/2, and ELK (Fig. 4). In an accompanying study, we showed that cell adhesion to AGGF1 was not disrupted by neutralizing antibodies against integrins α1, α2, α3, α4, α6, αv, and β3; therefore, these integrins are unlikely to affect the function of AGGF1 in VSMCs. However, we found that integrin α5β1 acted as a receptor for AGGF1 in endothelial cells, activated FAK-Src-AKT signaling, and modulated the signaling and functions of AGGF1 in endothelial cells and therapeutic angiogenesis in a model for peripheral artery disease (PAD) (16). The question is whether integrin α5β1 also acts as a receptor for AGGF1 in VSMCs. One published study showed that integrin α5 had no effect on the proliferation and migration of VSMCs (25). We showed that knockdown of integrin α5 affected the phenotypic switching of VSMCs by reducing the expression of smooth muscle contractile markers (Fig. S3*B*). However, this effect could not be explained by the finding that knockdown of integrin α5 decreased the phosphorylation of MEK1/2, ERK1/2, and ELK (Fig. S4*B*). Moreover, siITGA5 did not have much effect on the functions of AGGF1 that significantly increased the expression of smooth muscle contractile markers and inhibited activation of MEK1/2, ERK1/2 and ELK (Figs. S3*B* and S4*B*). The data suggest that AGGF1 regulates VSMC functions independent of integrin α5. It is not clear why integrin α5 does not act as an AGGF1 receptor on VSMCs. One possible explanation may be the contrasting effects that knockdown of integrin α5 decreased the phosphorylation of MEK1/2, ERK1/2, and ELK (Fig. S4*B*), whereas knockdown of integrin α7 increased the phosphorylation of MEK1/2, ERK1/2, and ELK (Fig. 5, *C* and *D*). Moreover, although VSMCs have both integrin α7 and integrin α5 on their surface, the interaction of AGGF1 is more competitive with integrin α7 than with integrin α5, and role of integrin α5 may be masked by integrin α7.

There are limitations with the present study. First, our data suggest that integrin α7 is a functional receptor for AGGF1 on VSMC surface; however, we cannot exclude the possibility that some other proteins may also act as potential receptors for AGGF1 to regulate VSMC functions. Second, in addition to integrin α7, AGGF1 may regulate VSMC functions through other regulatory factors and biological pathways. For example, Sanz-González *et al.* (26) showed that p53 overexpression transgenic mice under *ApoE* KO background showed attenuated neointimal formation in mechanically injured femoral arteries. We recently reported that AGGF1 increased p53 stability by interacting with p53, increasing phosphorylation and acetylation of p53, and inhibiting p53 ubiquitination (27). Therefore, AGGF1 may inhibit neointimal formation after vascular injury through increased p53 expression.

In conclusion, our data identify integrin α7 as a cell surface receptor for AGGF1 on VSMCs and suggest that AGGF1 regulates VSMC phenotypic switching, proliferation, and migration by interacting with integrin α7 through a functional domain between amino acids 564 to 614. The deletion of the functional AGGF1 domain abrogated the functions of AGGF1 in inhibition of MEK-ERK1/2-ELK signaling, upregulation of contractile markers α-SMA and SM22, inhibition of VSMCs proliferation and migration, and regulation of key genes involved in cell cycle regulation and proliferation, and attenuation of neointimal formation in mouse carotid arteries after vascular injury. Moreover, we show that integrin α7 is required for AGGF1 functions as knockdown of expression of *ITGA7*, but not *ITGA8* or *ITGA5*, reversed the function of AGGF1 in inhibiting MEK-ERK1/2-ELK signaling. The results provide deep understanding of the molecular mechanisms for a novel AGGF1-based therapy for neointimal formation and restenosis after vascular injury.

## Experimental procedures

### Plasmids

Serial N-terminal and C-terminal deletion mutants of *AGGF1* were generated using pET-28b-AGGF1 as the template (5). The primers designed to create these mutants are shown in Tables S1 and S2. A total of 12 N-terminal deletions and 13 C-terminal serial deletion constructs of *AGGF1* were generated by PCR and subcloning. Each plasmid was transformed to *E. coli* BL21 (DE3) with a T7 RNA Polymerase-based system for protein expression. The 6 × His-tagged wild type (WT) AGGF1 (AGGF1-WT) and serial deletion mutants (AGGF1-N1–N12 and AGGF1-C1–C13) were purified as described by us previously (28). The purified protein was measured by an Enhanced BCA Protein Assay Kit (P0010, Beyotime).

The luciferase reporters with the promoter sequences of three VSMCs phenotypic switching markers cloned into pGL3-luc promoter vector (Promega), including MYH11-luc, α-SMA-luc, and SM22-luc, were described previously (6). The open reading frame of the *SRF* gene was subcloned into pGFP-N1 (pGFP-SRF) as described (6).

### Cell culture and transfection

MOVAS-1, an immortalized mouse aorta VSMC line, was purchased from ATCC (American Type Culture Collection). MOVAS-1 cells were maintained in the high-glucose Dulbecco’s Modified Eagle’s medium (DMEM) supplemented with 10% (V/V) fetal bovine serum (FBS, Gibico Life Technologies) in a humidified water jacket incubator with 5% CO_2_ at 37 °C.

Small interfering RNA against *ITGA7* (siITGA7) and *ITGA8* (siITGA8) and a negative control siRNA (siNC) were purchased from RioboBio. Transfection of VSMCs with siRNA (100 nmol) or an expression plasmid was carried out using Lipofectamine RNAiMAX according to manufacturer’s instructions (Thermo Fisher 13778030).

### Western blotting

Western blot analysis was carried out as described previously (28). Protein extracts from cultured MOVAS-1 cells or mouse carotid artery samples were prepared with Western-IP lysis buffer (Beyotime) supplemented with proteinase inhibitor cocktail (Roche). The primary antibodies used for Western blotting include: anti-AGGF1 (Proteintech, 1189-1, 0.7 μg/ml), anti-MYH11 (Proteintech, 21404-1, 0.7 μg/ml), anti-α-SMA (Proteintech, 55135-1, 0.6 μg/ml), anti-SM22 (Proteintech, 10493-1, 0.33 μg/ml), anti-GAPDH (Proteintech, 60004-1, 0.166 μg/ml), anti-pMEK1/2 (Bioss, bsm-52176R, 1 μg/ml), anti-pELK (Bioss, bs-10154R, 1 μg/ml), anti-ERK1/2 (Abcam, 184699, 1.2 μg/ml), anti-pERK1/2 (Abcam, 223500, 1:1000 dilution), anti-integrin α5 (Proteintech, 10569-1, 0.5 μg/ml), anti-integrin α7 (Abclonal, A14246, 1:500), and anti-integrin α8 (Abclonal, A13056, 1:500). The secondary antibodies include a goat anti-rabbit IgG or a goat anti-mouse IgG (HRP-conjugated, 1:20,000) from Biofly. Images from Western blot analysis were captured using a ChemiDoc XRS (Bio-Rad Laboratories) with the SuperSignal West Pico Chemiluminescent Substrate (Pierce Chemical Co) and further analyzed with Gel-Pro analyzer.

### Real-time PCR analysis

Quantitative real-time PCR analysis was performed as described previously (29). Total RNA was isolated from cells using TRIzol reagent (TaKaRa Biotech) and converted into cDNA with M-MLV reverse transcriptase (Promega). DNase I (Promega) was used to remove contaminating genomic DNA before reverse transcription. Quantitative PCR analysis was carried out using the FastStart Universal SYBR Green Master (Roche) on a 7900 HT Fast Real-Time PCR System (ABI). The PCR profile was 94 °C for 5 min, and 40 cycles of 94 °C for 10 s and 60 °C for 15 s. *GAPDH* was used as internal control. Each experiment was performed in triplicate and repeated at least three times.

### Cell proliferation assays

MOVAS-1 cells were seeded onto 96-well plates, cultured for 36 h, and subjected to proliferation analysis using a CCK-8 kit (Dojindo Laboratories) according to the manufacturer’s protocols. Cell proliferation was measured with readings at the absorbance of 450 nm with a microplate reader. Cell proliferation assays were also performed using EdU staining with an EdU kit (BeyoClick EdU Cell Proliferation Kit with Alexa Fluor 488, Beyotime). MOVAS cells were seeded in 24-well plates, cultured for 18 h, and treated with wild-type or mutant AGGF1 *versus* control PBS for 4 h. Subsequently, cells were incubated with EdU for 2 h, fixed with 4% paraformaldehyde for 15 min, photographed, and counted.

### Cell migration assays

Cell migration assays were performed as described (6). MOVAS cells were starved in serum-free DMEM for 6 h and then grown in 6-well plates with 1.5 ml of DMEM/10% FBS as a confluent monolayer (overnight). A mechanical scratch was then made with a pipette tip at the bottom of the well. The cells were then incubated with 20 μl of 5 μg/ml wild-type or mutant AGGF1 *versus* 20 μl control PBS and allowed to migrate for 24 h. Images were captured under a Nikon ECLIPSE Ti microscope and analyzed using Image-Pro Plus 6.0. Cell migration was quantified as (cell free area at 0 h – cell free area at 24 h)/cell free area at 0 h.

Cell migration was also examined in Transwells in Boyden chambers. Transwells were rinsed in a Petri dish containing PBS, washed with serum-free DMEM, filled with serum-free DMEM (500 μl), and placed in a 37 °C incubator before experiments. MOVAS cells were starved in serum-free DMEM for 6 h and adjusted to a concentration of 1 × 10^5^ cells/ml. DMEM/10% FBS (500 μl) was added to the lower chamber of Boyden chambers. The medium was removed from Transwells, which were then placed into Boyden chambers. In total, 100 μl of cell suspension was added to the Transwells. Cells were then treated with 20 μl of 5 μg/ml wild-type or mutant AGGF1 *versus* 20 μl control PBS for 12 h. Transwells were carefully removed from the chamber, washed twice with PBS, fixed with methanol for 30 min, and dried thoroughly. Transwells were then treated in a crystal violet dye solution for 20 min, rinsed with double distilled water, photographed, and counted.

### Cell cycle assays

Cell cycle analysis was carried out with a Cell Cycle and Apoptosis Detection kit (C1052, Beyotime) as described previously (6). MOVAS cells were trypsinized, pelleted by centrifugation at 1000*g* for 3 to 5 min, washed with 1 ml of ice-cooled PBS, and placed in precooled 70% ethanol in an ice bath for 12 h. Cells were pelleted by centrifugation at about 1000*g* for 3 to 5 min, washed with 1 ml of ice-cooled PBS, and incubated with 0.5 ml of a propidium iodide staining solution at 37 °C in the dark for 30 min. Cells were then analyzed by flow cytometry.

### Cell adhesion assays

Wild-type or mutant AGGF1 protein (20 μl of 5 μg/ml) was added to a 96-well plate and placed at 37° for 1 h. The wells were then washed with D-PBS three times to remove unbound proteins, blocked with D-PBS containing 1% BSA at 37 °C for 1 h, and washed once with D-PBS. Serum-starved MOVAS cells were pelleted by centrifugation and resuspended in HBSS. In total, 150 μl of cells (3 × 10^5^ cells/ml) was added to each AGGF1-coated well of a 96-well plate and incubated for 1 h. Unbound cells were washed away with PBS, and bound cells were photographed and counted.

### Dual luciferase reporter assay

MOVAS-1 cells were washed with PBS (Phosphate Buffer Saline) twice, lysed using passive lysis buffer (Promega) at room temperature for 20 min, and centrifuged at 13,000 rpm for 10 min. A total of 10 μl of clear supernatant was transferred to a new Eppendorf tube for luciferase assays. Firefly and Renilla luciferase activities were measured using the Dual-Glo luciferase assay kit (Gibico Life Technologies) and Glomax20/20 (Promega) as described by us previously. Each experiment was performed in triplicate and repeated at least three times.

### Co-immunoprecipitation (Co-IP)

Co-IP studies were carried out as described previously (30). In brief, HeLa cells were cultured to ∼80% confluence in a 10-cm plate (1 × 10^7^ in 7 ml media) and transfected with 10 μg of plasmid DNA. The cell lysate was preabsorbed with 30 μl of Protein A/G PLUS-agarose for 1 h at 4 °C and microcentrifuged at 4 °C. An equal volume of the supernatants was incubated with 1.5 μg of an immunoprecipitation antibody or the same amount of anti-mouse IgG on a rotator overnight at 4 °C and then mixed with 30 μl of Protein A/G PLUS-agarose. Precipitated proteins were resolved by 10% SDS-PAGE gel electrophoresis and then immunoblotted with an antibody for the target protein.

### Preparation of shRNA lentiviruses

We cloned a DNA fragment encoding shRNA for *ITGA7* or control shNC into the lentiviral vector pLVX-shRNA2-Puro. We cotransfected the viral plasmid and the helper plasmids pMD2G and psPAX2 at a ratio of 2:1:1 into 293T cells, collected the cell supernatant after 48 h of transfection, and tested the titers after concentration and purification. Lentiviruses were injected to mice through a tail vein. The titer of the lentivirus was adjusted to 5 × 10^8^ TU/ml, and 60 μl of the virus stock (*i.e.*, 3 × 10^7^ virus particles) per mouse was injected. Two weeks after the virus injection, the mice were used for creating the model for neointima formation after vascular injury and treated with AGGF1 *versus* PBS as described below. Twenty-eight days after the surgery, the mice were anesthetized, sacrificed, and used for follow-up studies.

### Wire-induced vascular injury of the carotid artery

C57BL/6N wild-type mice (8–10 weeks of age) were used in our studies. Both male and female mice were maintained in an SPF animal room, and appropriate temperature (22 ± 2 °C), humidity (55 ± 5%), and 12 h/12 h dark/light cycle were maintained. Animal care and experimental procedures were approved by the Ethics Committee on Animal Research at Huazhong University of Science and Technology and performed in accordance with institutional and NIH guidelines.

The carotid artery restenosis model was created by guidewire injury with male mice at the age of 10 to 12 weeks (20–25 g). Mice were anesthetized with an intraperitoneal injection of sodium pentobarbital (50 mg/kg) prior to vascular injury. The left common carotid artery was injured by inserting and removing a 0.014-in guidewire three times. We used the right carotid artery that was isolated but not injured as the Sham group. For protein administration, the mice were randomly divided into appropriate groups and injected intravenously with purified proteins (100 μl of 1.2 μM AGGF1-WT, AGGF1-ADD, AGGF1-C1, AGGF1-C2 or AGGF1-C3) or the same amount of PBS (n = 6/group) 1 day after vascular injury, twice a week for 4 weeks.

### Immunostaining

Consecutive frozen sections of carotid arteries were immunostained with an anti-SM22and α-SMA antibody and then incubated with a biotinylated secondary antibody as described previously (6). Sections were also counterstained with hematoxylin and eosin as described (28). The intensity of the immunostaining signal was determined by measurements of the integrated optical density with light microscopy using a computer-based Image-Pro System as described previously (28).

### Data analysis

All experiments were repeated at least three times. The data were presented as mean ± SD. Two-group comparisons were analyzed by the two-tailed, paired, or unpaired Student’s *t* test. A one-way ANOVA with Dunnett post hoc test was used to compare multiple groups. *p* < 0.05 was considered to be statistically significant. Data were analyzed using GraphPad Prism 8.0.

## Data availability

The authors declare that all supporting data are available within the article and the Supporting Information.

## Supporting information

This article contains supporting information.

## Conflict of interest

The authors declare that they have no conflicts of interest with the contents of this article.
