# Supplementary material for: Angiogenic factor AGGF1 blocks neointimal formation after vascular injury via interaction with integrin α7 on vascular smooth muscle cells
Source: J Biol Chem. 2022 Feb 22;298(4):101759. doi: 10.1016/j.jbc.2022.101759 (PMC8968644; doi:10.1016/j.jbc.2022.101759)
Supplement: Supplemental Figures S1–S7, Tables S1 and S2 [file mmc1.doc]

**Supporting Information**

**Angiogenic factor AGGF1 Blocks Neointimal Formation after Vascular Injury via Interaction with Integrin α7 on Vascular Smooth Muscle Cells**

**Yubing Yu^1,3^, Yong Li^2,3^, Huixin Peng^1,3^, Qixue Song^1,3^, Xingwen Da^1^, Hui Li^1^, Zuhan He^1^, Xiang Ren^1^, Chengqi Xu^1,*^, Yufeng Yao^1,*^, and Qing K. Wang^1,*^**

^1^Key Laboratory of Molecular Biophysics of the Ministry of Education, College of Life Science and Technology and Center for Human Genome Research，Huazhong University of Science and Technology, Wuhan, P. R. China

^2^College of Biotechnology, Guilin Medical University

^3^These authors contributed equally to this work.

Correspondence to: Yufeng Yao, Chengqi Xu, or Qing K. Wang, Center for Human Genome Research and College of Life Science and Technology, Huazhong University of Science and Technology, 1037 Luoyu Road, Wuhan, P. R. China.

Email: yaoyfeng@hust.edu.cn, cqxu@mail.hust.edu.cn, or qingwang118@qq.com

**Supplemental Figures**

**
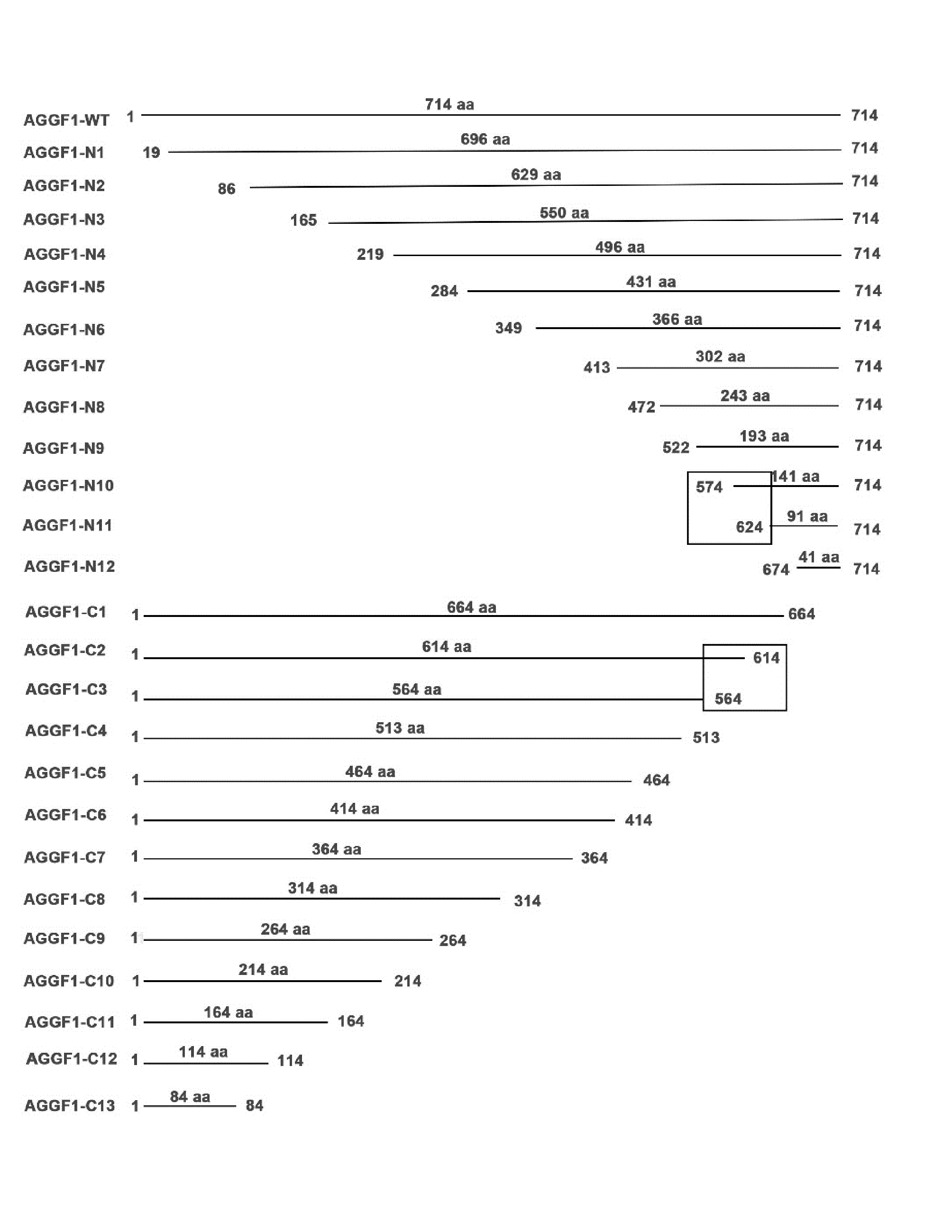
**

**Figure S1.** **Schematic diagram showing the structure of wild type (WT) and serial N-terminal and C-terminal deletion mutants of AGGF1.**

The starting amino acid positions of deletions N1 to N12 and the ending amino acid positions of C1 to C13 are marked with numbers.


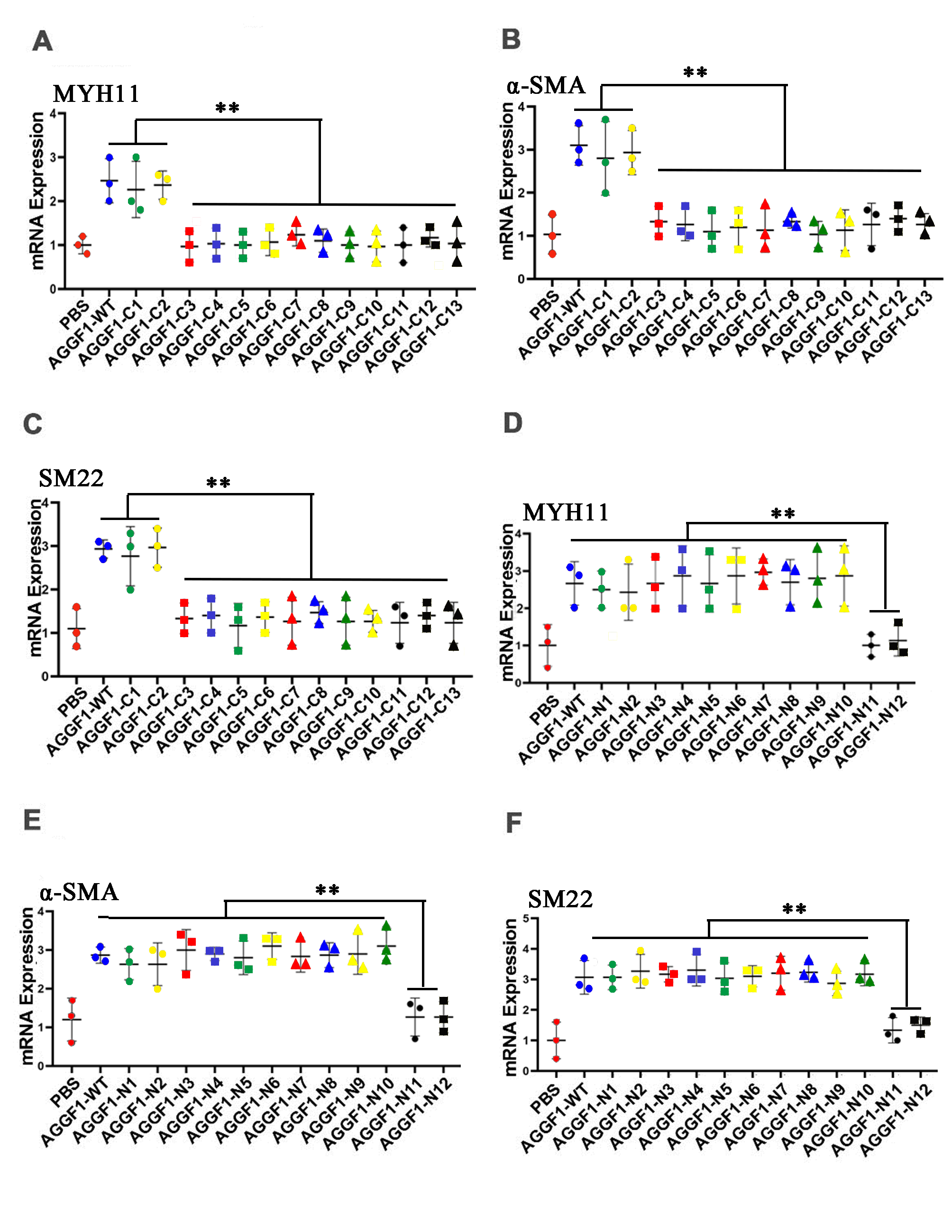


**Figure S2. RT-PCR analysis of markers MYH11, α-SMA and SM22 for** **phenotypic switching of VSMCs defined the critical AGGF1 functional domain between C2 and C3 (amino acids 614-564) and between N10 and N11 (amino acids 574-624).**

Total RNA was isolated from MOVAS-1 cells treated with 5ug/ml of WT or mutant AGGF1 for 24h, and used for real-time RT-PCR analysis. PBS was used as a negative control.

**(A)**, **(B)** and **(C)**, Effects of WT AGGF1 and C-terminal deletions on expression of MYH11, α-SMA and SM22. **(D)**, **(E)** and **(F)**, Effects of WT AGGF1 and N-terminal deletions on expression of MYH11, α-SMA and SM22. Data are shown as mean±SD. ***P*<0.01, n=3/group (one-way ANOVA with Dunnett test for multiple comparison).


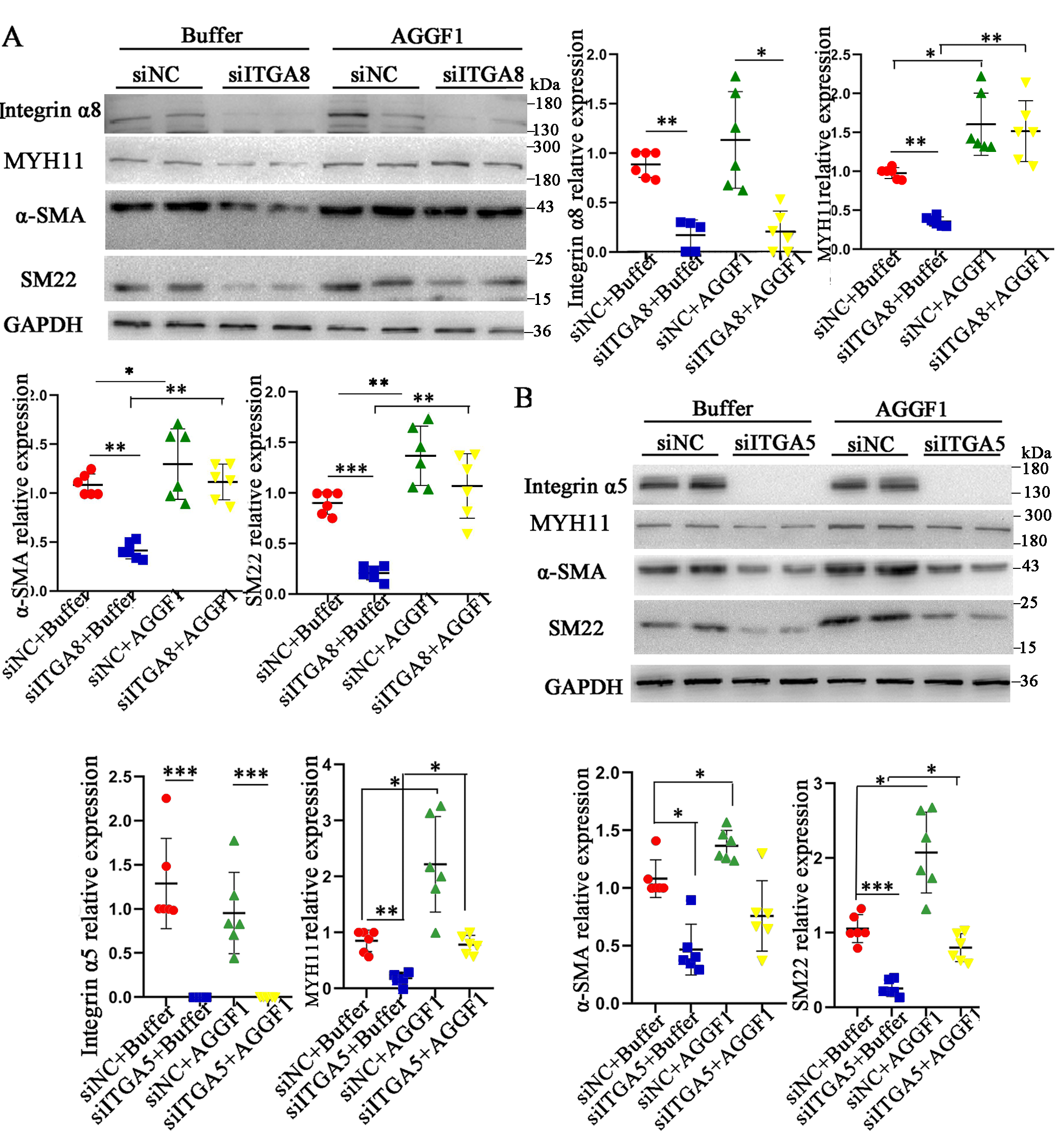


**Figure S3. AGGF1 regulates phenotypic switching of VSMCs independent of *ITGA5* or *ITGA8*.**

**(A)**, Western blot analysis showed that knockdown of *ITGA8* did not affect the ability of AGGF1 to enhance the expression of MYH11, α-SMA and SM22 in MOVAS cells. MOVAS cells were transfected with control siNC or *ITGA8* siRNA (siITGA8), and incubated with 20 μl control PBS or 20 μl of WT AGGF1 (5 μg/ml) for 24 hours, lysed, and used for Western blot analysis (mean±SD, one-way ANOVA with Dunnett test for multiple comparison; **P*<0.05, ***P*<0.01, n=6/group). **(B),** Western blot analysis showed that AGGF1 enhanced the expression of MYH11, α-SMA and SM22 in MOVAS cells, and the effect was not affected by knockdown of *ITGA5* by siITGA5. MOVAS cells were treated and used as in (**A**). Data are shown as mean±SD (one-way ANOVA with Dunnett test for multiple comparison; **P*<0.05, ***P*<0.01, ****P*<0.001, n=6/group).


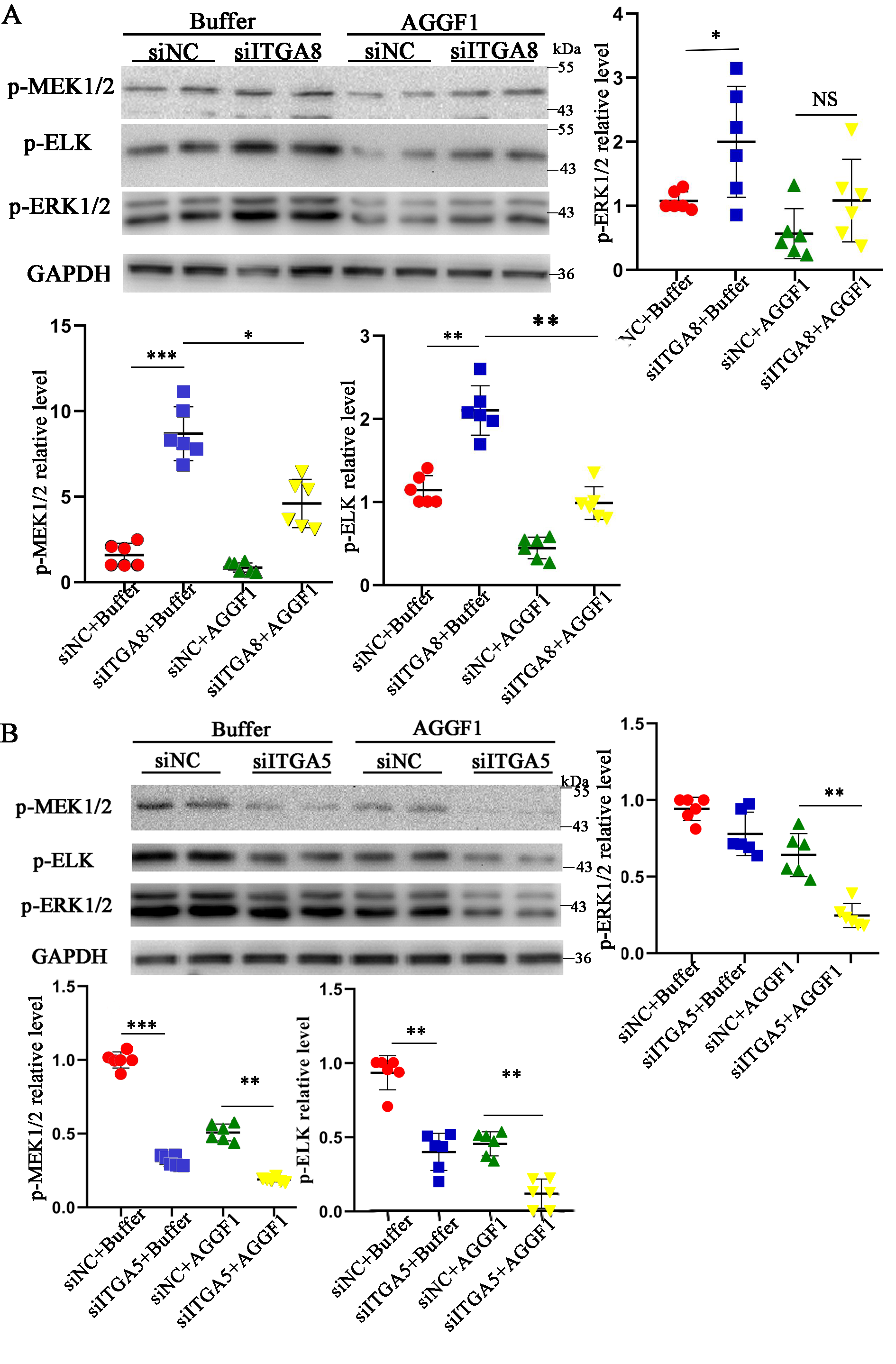


**Figure S4. AGGF1-mediated inhibition of phosphorylation of MEK and ERK1/2 was not affected by knockdown of *ITGA5* or *ITGA8*. (A, B)** Western blot analysis for phosphorylated MEK1/2, ERK1/2 and ELK. MOVAS cells were transfected with control siNC, *ITGA5* siRNA (siITGA5), or *ITGA8* siRNA (siITGA8), and incubated with 20 μl control PBS or 20 μl of WT AGGF1 (5 μg/ml) for 15 minutes, lysed, and used for Western blot analysis (mean±SD, one-way ANOVA with Dunnett test for multiple comparison; **P*<0.05, ***P*<0.01, ****P*<0.001, n=6/group).


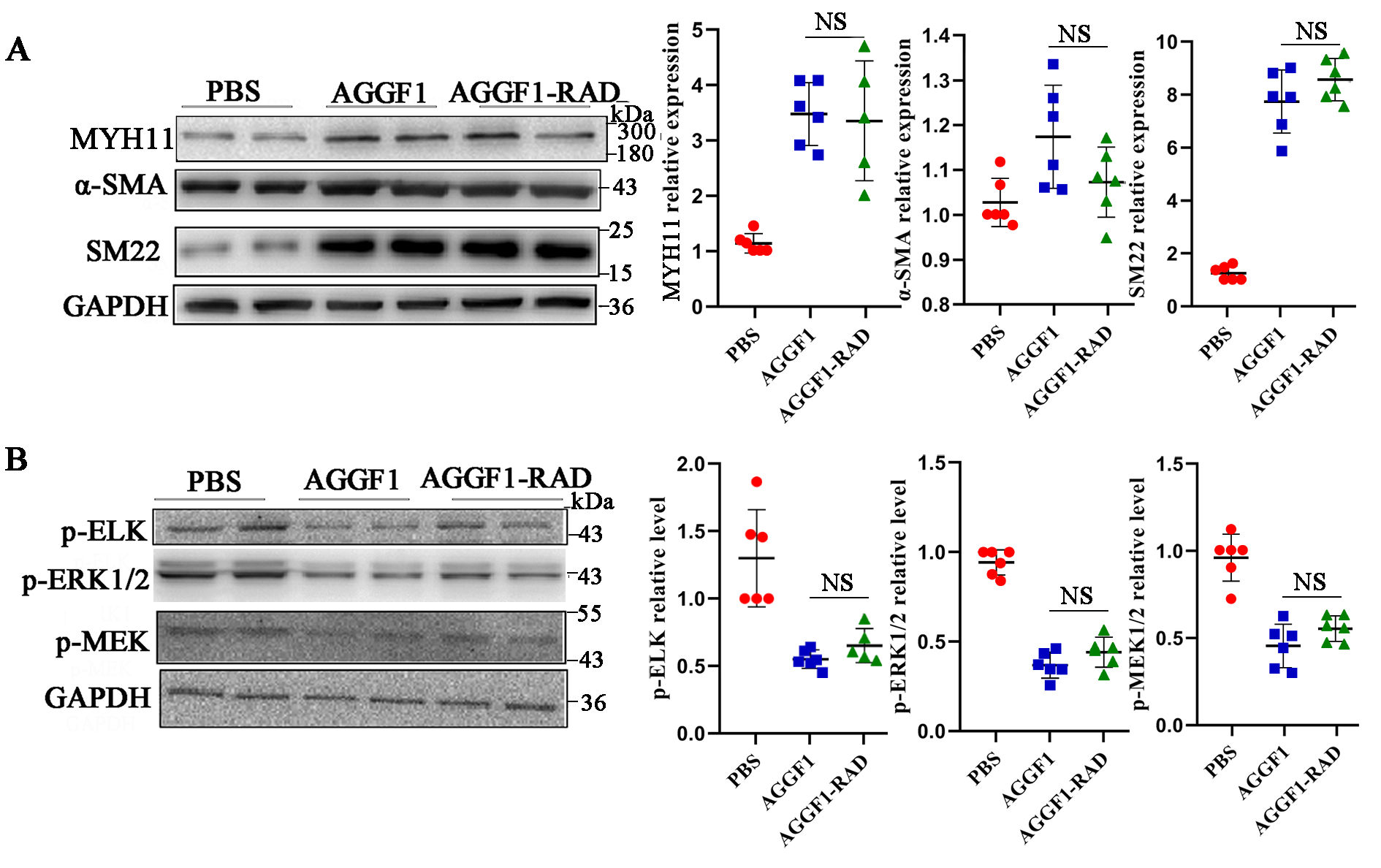


**Figure S5. Effect of mutant AGGF1-RAD on functions of smooth muscle cells. (A),** Western blot analysis showed that AGGF1 enhanced the expression of MYH11, α-SMA and SM22, but the effect was not affected the AGGF1-RAD mutation. MOVAS cells were treated with 20 μl control PBS or 20 μl of wild type AGGF1 or mutant AGGF1-RAD (5 μg/ml) for 24 hours, lysed, and used for Western blot analysis (mean±SD, one-way ANOVA with Dunnett test for multiple comparison; NS, not significant, n=6/group. **(B)**, Western blot analysis showed that AGGF1 inhibited phosphorylation of ELK, MEK and ERK1/2, and the effect was not affected by the AGGF1-RAD mutation. MOVAS cells were as in (**A**) but for 15 minutes, lysed, and used for Western blot analysis **(**mean±SD, one-way ANOVA with Dunnett test for multiple comparison; NS, not significant, n=6/group).


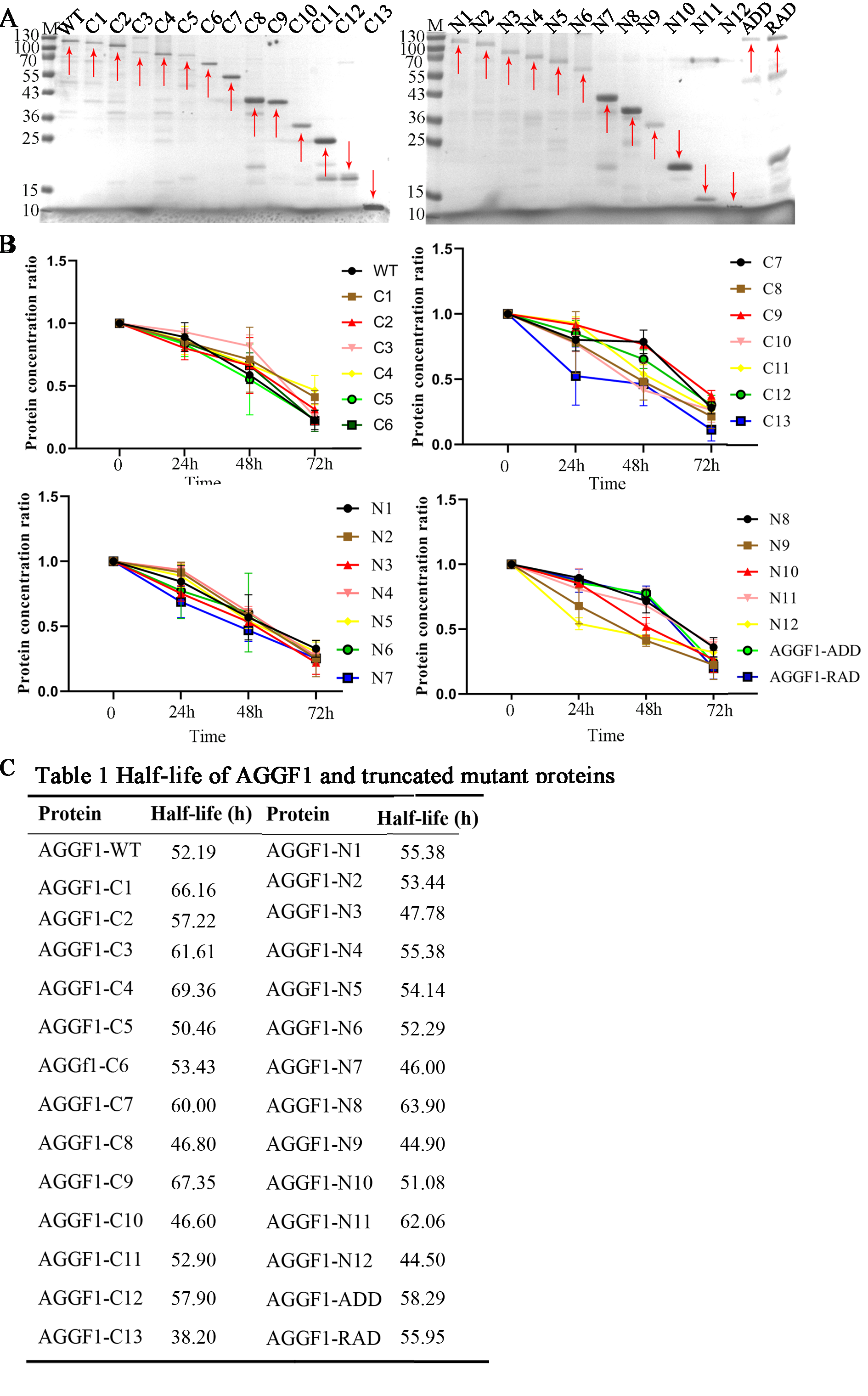


**Figure S6. Stability of purified wild type and mutant AGGF1 proteins.**

**(A),** Wild type (WT) and mutant AGGF1 (C1-C13, N1-N12, ADD and RAD) were purified from *E. coli* using a nickel bead gel kit, separated by 12% SDS-PAGE, and visualized by Coomassie brilliant blue staining. **(B),** AGGF1 protein stability assays. The concentration of each purified AGGF1 protein was measured by a BCA kit. The same volume of AGGF1 (80 μl) was placed in a constant temperature incubator at 37℃, and the changes of the protein concentrations were monitored at different time points of 0 h, 24 h, 48 h and 72 h. The AGGF1 concentrations were plotted against time points. The concentration at time point 0 was set at 1.0 for each protein, and the concentrations at other time points were calibrated with that at 0 h. The time point at which the AGGF1 concentration was reduced by 50% was extrapolated from each graph and considered as the half-life of each protein. **(C),** Half-life of wild type and mutant AGGF1. n= 3/group.


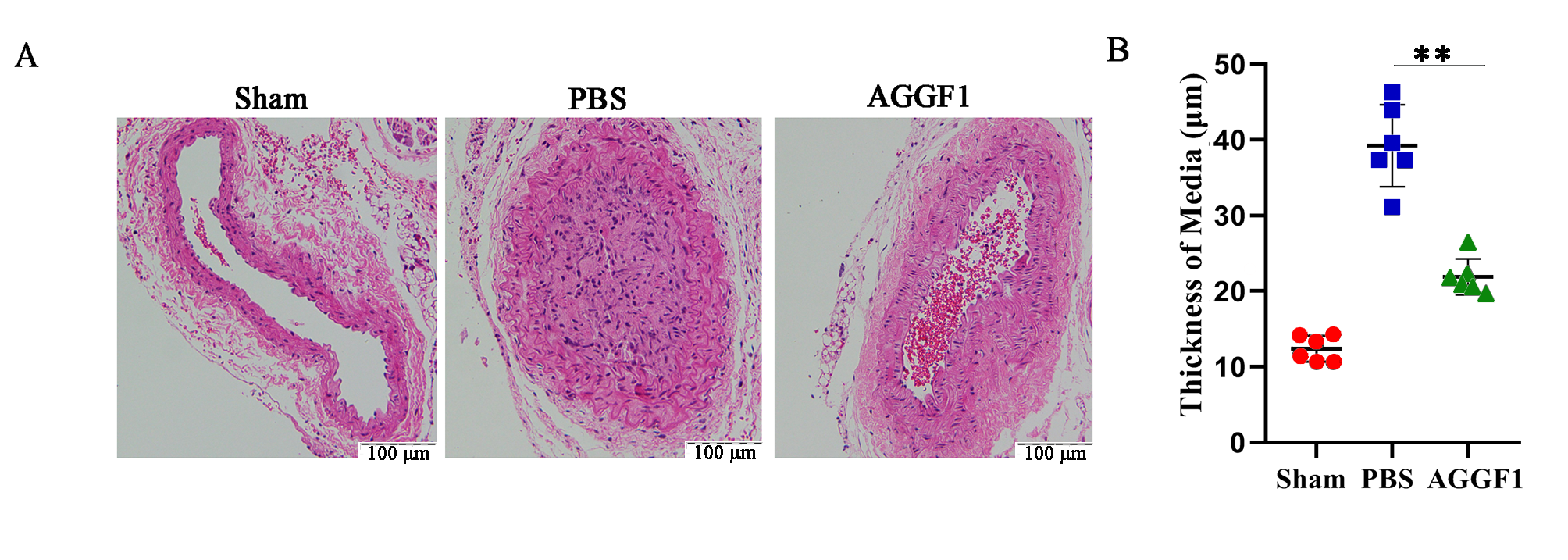


**Figure S7. AGGF1-WT protein treatment successfully blocked neointimal formation after vascular injury in female mice.**

**(A)**, Representative H&E staining images are shown. **(B)**, H&E staining images were quantified and plotted. ***P*<0.01, n=6/group (Student’s *t* test).

**Supplemental Tables**

**Table 1. Primers used for construction of serial N-terminal *AGGF1*** deletion mutatis.

| **Primers** | **Sequences (5’ to 3’)** | **Deleted Amino Acids**  **Acids Residues** |
| --- | --- | --- |
| AGGF1 N1 F | ATTG GAT CCC GAG CCT GAG CTG GCC CAG | Δ 1-18 |
| AGGF1 N2 F | ATTG GAT CCC AAT AAA AAG TCT GAT GTA GAA | Δ 1-85 |
| AGGF1 N3 F | ATTG GAT CCC GAC CAT TTT GCC TCA AAT TCA | Δ 1-164 |
| AGGF1 N4 F | ATTG GAT CCC CAC AGC ACT GGT TTC TAT TAT | Δ 1-218 |
| AGGF1 N5 F | ATTG GAT CCC TCT GCA ACA AAT GAG GAA AAG | Δ 1-283 |
| AGGF1 N6 F | ATTG GAT CCC AAC ATC TCT AAT TCA ACA TCA | Δ 1-348 |
| AGGF1 N7 F | ATTG GAT CCC ATT GTC ATT AGA TCA CCT GTG | Δ 1-412 |
| AGGF1 N8 F | ATTG GAT CCC GTG GAT CAA GGC AGT CAA AAT | Δ 1-471 |
| AGGF1 N9 F | ATTG GAT CCC ACC TGT GAT GGA TGT GAA | Δ 1-521 |
| AGGF1 N10 F | ATTG GAT CCC ACA GAA TAC GAA GAT GAA AAG | Δ 1-573 |
| AGGF1 N11 F | ATTG GAT CCC CGG AAG ATG TTG GAG AAG ATG | Δ 1-623 |
| AGGF1 N12 F | ATTG GAT CCC CTC CAA AAC AAG AAC AAA AAA | Δ 1-673 |
| AGGF1 N R | TAA AGC GGC CGC TCA CTC TAA AGT CCC TTT TAC CCA |  |

| **Primers** | **Sequences (5’ to 3’)** | **Deleted Amino Acids** |
| --- | --- | --- |
| AGGF1 C1 F | TATA GC GGC CGC TCA TTT GCC TGT CCC CAA | Δ 665-714 |
| AGGF1 C2 F | TATA GC GGC CGC TCA ATG AAC AGA TGC AGG | Δ 615-714 |
| AGGF1 C3 F | TATA GC GGC CGC TCA TTT CTT TAA TTC TTT | Δ 565-714 |
| AGGF1 C4 F | TATA GC GGC CGC TCA AAA GGA TAA GAC AGT | Δ 514-714 |
| AGGF1 C5 F | TATA GC GGC CGC TCA ATG GTC AAA ATA AAT | Δ 465-714 |
| AGGF1 C6 F | TATA GC GGC CGC TCA GAC AAT TAC TCT AAT | Δ 415-714 |
| AGGF1 C7 F | TATA GC GGC CGC TCA AGT CTC CAT GAT TTT | Δ 365-714 |
| AGGF1 C8 F | TATA GC GGC CGC TCA TGC GAA ATT TTC TTC | Δ 315-714 |
| AGGF1 C9 F | TATA GC GGC CGC TCA AGA AGT CGG ATA AGG | Δ 265-714 |
| AGGF1 C10 F | TATA GC GGC CGC TCA TCC AGT ATT TTC ATC | Δ 215-714 |
| AGGF1 C11 F | TATA GC GGC CGC TCA CAC TTG TCT ATA TTT | Δ 165-714 |
| AGGF1 C12 F | TATA GC GGC CGC TCA GTC ATT GTA GTA CGT | Δ 115-714 |
| AGGF1 C13 F | TATA GC GGC CGC TCA TTC ATT TCT CCC ACG | Δ 85-714 |
| AGGF1 C R | AA TTG GAT CCG ATG GCC TCG GAG GCG CCG |  |

**Table 2.Primers used for construction of serial C-terminal *AGGF1* deletion mutants.**
